# Environmental purines decrease *Pseudomonas aeruginosa* biofilm formation by disrupting c-di-GMP metabolism

**DOI:** 10.1016/j.celrep.2024.114154

**Published:** 2024-04-25

**Authors:** Corey Kennelly, Peter Tran, Arthur Prindle

**Affiliations:** 1 Department of Biochemistry and Molecular Genetics, Feinberg School of Medicine, Northwestern University, Chicago, IL 60611, USA; 2 Center for Synthetic Biology, Northwestern University, Evanston, IL 60208, USA; 3 Department of Chemical and Biological Engineering, Northwestern University, Evanston, IL 60208, USA; 4 Lead contact

## Abstract

Cyclic di-guanosine monophosphate (c-di-GMP) is a bacterial second messenger that governs the lifestyle switch between planktonic and biofilm states. While substantial investigation has focused on the proteins that produce and degrade c-di-GMP, less attention has been paid to the potential for metabolic control of c-di-GMP signaling. Here, we show that micromolar levels of specific environmental purines unexpectedly decrease c-di-GMP and biofilm formation in *Pseudomonas aeruginosa*. Using a fluorescent genetic reporter, we show that adenosine and inosine decrease c-di-GMP even when competing purines are present. We confirm genetically that purine salvage is required for c-di-GMP decrease. Furthermore, we find that (p)ppGpp prevents xanthosine and guanosine from producing an opposing c-di-GMP increase, reinforcing a salvage hierarchy that favors c-di-GMP decrease even at the expense of growth. We propose that purines can act as a cue for bacteria to shift their lifestyle away from the recalcitrant biofilm state via upstream metabolic control of c-di-GMP signaling.

## In brief

Kennelly et al. demonstrate that adenosine and inosine decrease c-di-GMP and biofilm formation of *Pseudomonas aeruginosa*. Without (p)ppGpp, xanthosine and guanosine increase c-di-GMP, but adenosine and inosine block this increase. This work reveals that environmental purines can act as a cue to shift bacteria away from the recalcitrant biofilm state.

## Graphical Abstract

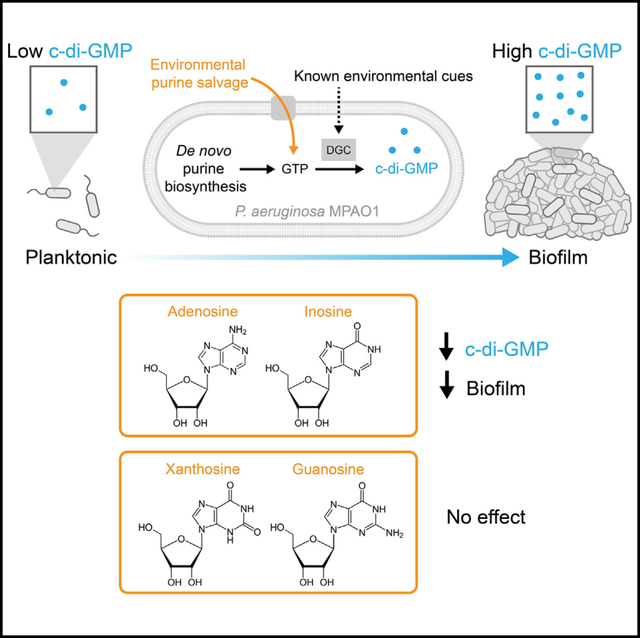

## INTRODUCTION

Bacteria predominately exist in densely packed surface-attached communities known as biofilms.^1^ Undesired bacterial biofilms present a challenging problem to human health, as biofilms readily form on liquid-exposed surfaces, can alter or degrade properties of these surfaces, can seed the propagation of pathogens, and resist treatment with antimicrobials and other perturbations.^2–4^ Furthermore, pathogenic biofilms are associated with nearly all human chronic wounds.^5^ *Pseudomonas aeruginosa* is a gram-negative opportunistic pathogen used as a model biofilm organism due to its propensity for biofilm formation and clinical relevance as one of the leading causes of nosocomial infection.^6^ In *P. aeruginosa* and many other bacteria, the second messenger cyclic di-guanosine monophosphate (c-di-GMP) drives biofilm formation by increasing production of exopolysaccharides and adhesins while downregulating flagellar-based motility, among other factors.^7^ Thus, understanding the cues that influence biofilm formation and dissolution via c-di-GMP could lead to improved control over undesired bacterial biofilms.

c-di-GMP levels can be affected by the activity of diguanylate cyclases, which produce c-di-GMP from guanosine triphosphate (GTP), and phosphodiesterases, which ultimately degrade c-di-GMP to guanosine monophosphate (GMP).^8–12^ The *P. aeruginosa* genome contains more than 40 genes encoding enzymes with domains associated with c-di-GMP synthesis or degradation.^7,13^ Several environmental cues that influence biofilm formation by acting on these enzymes have been identified. For example, membrane stress was shown to increase c-di-GMP levels by activating the diguanylate cyclase WspR, while nitric oxide can decrease c-di-GMP levels by activating the phosphodiesterases DipA and RbdA.^14,15^ A less appreciated hypothesis is that the levels of c-di-GMP are affected by changes in the concentration of the substrate GTP.^16^ GTP can be produced via *de novo* purine biosynthesis, and mutants lacking this pathway form aberrant biofilms.^17–19^ Additionally, azathioprine, which inhibits *de novo* purine biosynthesis, decreases c-di-GMP and biofilm formation in *E. coli* by disrupting the intracellular nucleotide pool.^20^ Thus, while many studies on c-di-GMP have focused on diguanylate cyclases and phosphodiesterases, less attention has been placed on the control of c-di-GMP signaling by precursor metabolism.

*P. aeruginosa* can salvage purines from the environment to conserve nutrients that would otherwise be spent on *de novo* purine biosynthesis.^17,21,22^ Here, we hypothesized that environmental purines may act as a cue to influence c-di-GMP and biofilm levels by directly interfacing with intracellular nucleotide pools. In this context, while it is commonly assumed that intracellular nucleotide pools remain in excess and are therefore irrelevant for nucleotide-derived second messenger signaling, we expected that the c-di-GMP precursors xanthosine and guanosine may increase c-di-GMP levels. However, we found that, counterintuitively, these c-di-GMP precursors had no effect, while the non-precursors adenosine and inosine decreased c-di-GMP and biofilm formation. This unexpected observation reveals that purines can act as a cue for bacteria to shift their lifestyle away from the recalcitrant biofilm state via upstream metabolic control of c-di-GMP signaling. Thus, when purine cues are present in the environment, *P. aeruginosa* appears to downregulate the persistent biofilm state thought to help tolerate nutrient limitation.

## RESULTS

### Adenosine and inosine decrease c-di-GMP and biofilm formation

**Figure 1.**
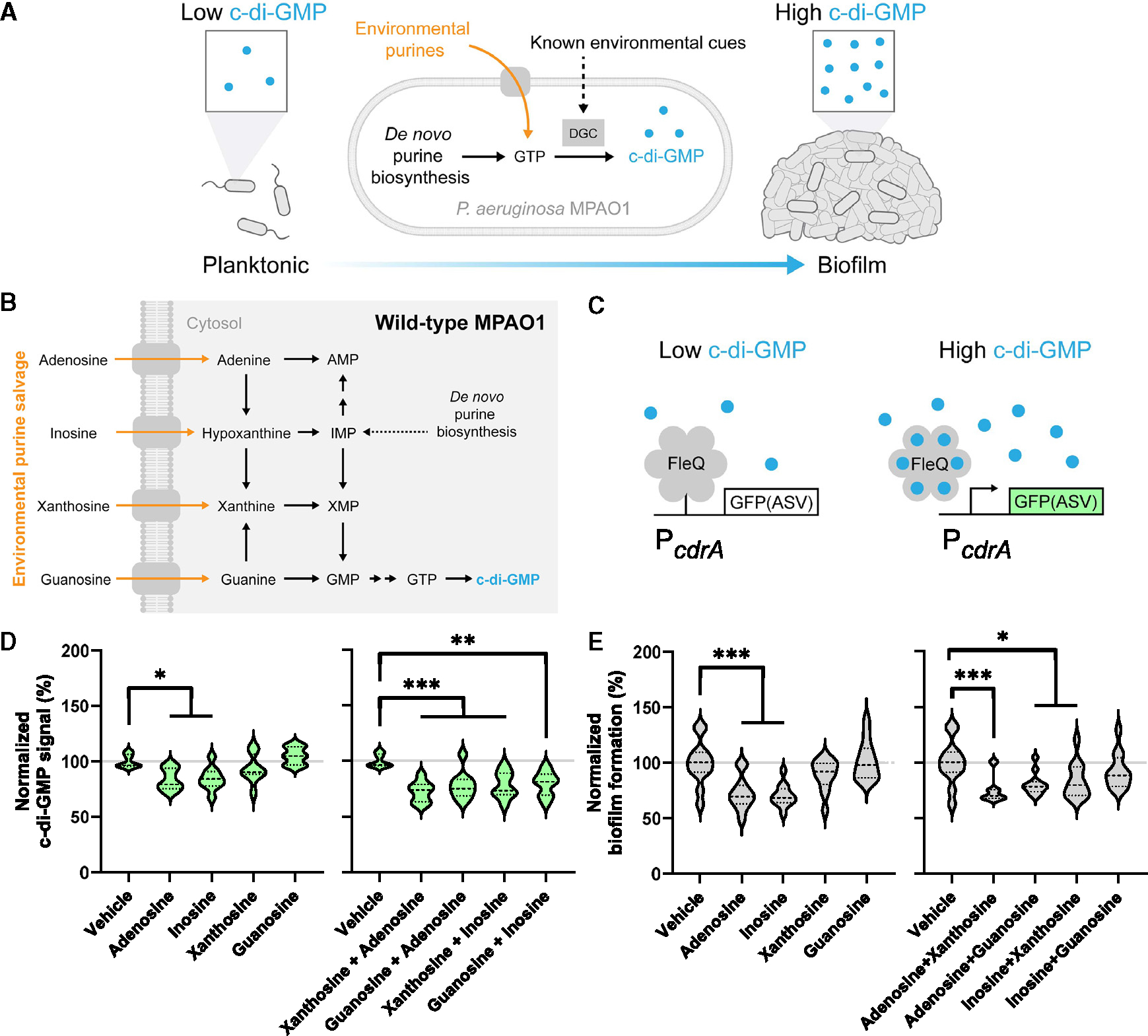
Adenosine and inosine decrease c-di-GMP and biofilm formation (A) The second messenger c-di-GMP is produced via GTP and positively influences biofilm state. Known environmental cues can affect c-di-GMP levels by altering the activity of diguanylate cyclases (DGCs) involved in c-di-GMP production. A largely unexplored hypothesis is that environmental purines may affect c-di-GMP levels via direct metabolic influence on c-di-GMP precursors. (B) Expanded purine metabolic pathway in *P. aeruginosa* for both *de novo* biosynthesis and salvage. (C) Fluorescent reporter pCdrA::GFP(ASV) functions via c-di-GMP binding that converts FleQ from repressing transcription to activating transcription. (D) Violin plots of c-di-GMP signal from pCdrA::GFP(ASV) normalized to OD_600_ growth for MPAO1 exposed to vehicle or 100 μM indicated compounds after 8 h of exposure at 37°C with shaking. 3 wells per condition per experiment from 3 independent experiments were included (*n* = 9), barring outliers removed due to aberrant growth: *n* = 8 (vehicle, xanthosine). All compounds were tested simultaneously. (E) Violin plots of biofilm formation from safranin-stained biomass at OD_530_ normalized to OD_600_ growth for MPAO1 exposed to vehicle or 100 μM indicated compounds after 8 h of exposure. 6 wells per condition per experiment from 2 independent experiments were included (*n* = 12). All compounds were tested simultaneously. *p ≤ 0.05, **p ≤ 0.01, and ***p ≤ 0.001. See also Figure S1.

Given that GTP is the substrate for c-di-GMP synthesis, we reasoned that exogenous purines may alter not only nucleotide levels as observed in early biochemical studies^23–27^ but also c-di-GMP levels and therefore biofilm formation (Figure 1A). Based on known *P. aeruginosa* purine metabolism (Figure 1B), we expected that direct precursor purines, such as guanosine, would increase abundance of c-di-GMP due to their ability to form GTP. To identify whether c-di-GMP levels change in response to exogenous purines in *P. aeruginosa*, we leveraged the well-established plasmid-based fluorescent reporter pCdrA::GFP(ASV).^28^ pCdrA::GFP(ASV) outputs more fluorescence when c-di-GMP levels are high and has a relatively fast turnover rate due to the ASV tag (Figure 1C). This experimental system allows us to test the hypothesis that environmental purines act as a cue for bacteria to regulate the biofilm state via c-di-GMP.

To test this hypothesis, we added the nucleosides adenosine, inosine, xanthosine, and guanosine at a concentration of 100 μM and tracked c-di-GMP signal. Unexpectedly, we found that adenosine and inosine decreased c-di-GMP, while xanthosine and guanosine had no effect (Figure 1D). Specifically, we observed that adenosine decreased c-di-GMP signal by 16.3% (*n* = 9) and that inosine decreased c-di-GMP signal by 15.6% (*n* = 9). We confirmed that this response is observed with their nucleobase equivalents, where adenine decreased c-di-GMP signal by 33.7% (*n* = 9) and hypoxanthine decreased c-di-GMP signal by 28.6% (*n* = 7) (Figure S1E). Since c-di-GMP is a second messenger that controls biofilm formation, we also quantified biofilm production using a safranin staining assay. Consistent with their effects on c-di-GMP, adenosine and inosine decreased biofilm formation, while xanthosine and guanosine had no effect (Figure 1E). Specifically, we found that adenosine reduced biofilm formation by 27.7% (*n* = 12) and that inosine reduced biofilm formation by 29.7% (*n* = 12). Adenosine and inosine also reduced biofilm formation relative to vehicle when added to established biofilms (Figure S1F). When we exposed other *P. aeruginosa* strains to these compounds, adenosine and inosine additionally reduced biofilm formation of strain PAK^29^ but not strain PA14^30^ (Figures S1H and S1I). Thus, our data suggest that c-di-GMP decrease, and the resulting biofilm formation decrease, is dependent on purine identity in a specific and unexpected manner rather than being a simple function of overall purine availability.

Because multiple purines may be present in the environment simultaneously, we also tested whether xanthosine and guanosine could block the effects of adenosine and inosine. We found that these pairwise mixed purines decreased c-di-GMP and biofilm formation to levels similar to those caused by adenosine or inosine alone (Figures 1D and 1E). We also tested the effect of an equimolar mixture of adenosine, inosine, xanthosine, and guanosine on c-di-GMP and found that this complete purine mixture also decreased c-di-GMP (Figure S1C). Thus, xanthosine and guanosine do not block the effects of adenosine and inosine. These results lead to the counterintuitive conclusion that purine precursors not only fail to increase c-di-GMP themselves but are also unable to compete with the inhibitory effects of more distant purines.

### The purine salvage pathway is required for the effects of adenosine and inosine

We next sought to identify the mechanism of how environmental purines lead to c-di-GMP decrease. First, we considered the possibility that a purine degradation product, such as uric acid, is the molecular intermediate in c-di-GMP signaling. Given that all purines share the same degradation pathway, the likelihood that a degradation product mediates their repressive effect seemed low. To rule out this possibility, we created a degradation-deficient mutant lacking xanthine dehydrogenase, Δ*xdhA*, which cannot catalyze the degradation of hypoxanthine to xanthine and xanthine to uric acid (Figure S2E). As expected, purine degradation is not required for adenosine and inosine to decrease c-di-GMP and biofilm formation (Figures S2G and S2H). Thus, our results suggest that an aspect of purine metabolism other than degradation is responsible for c-di-GMP decrease.

**Figure 2.**
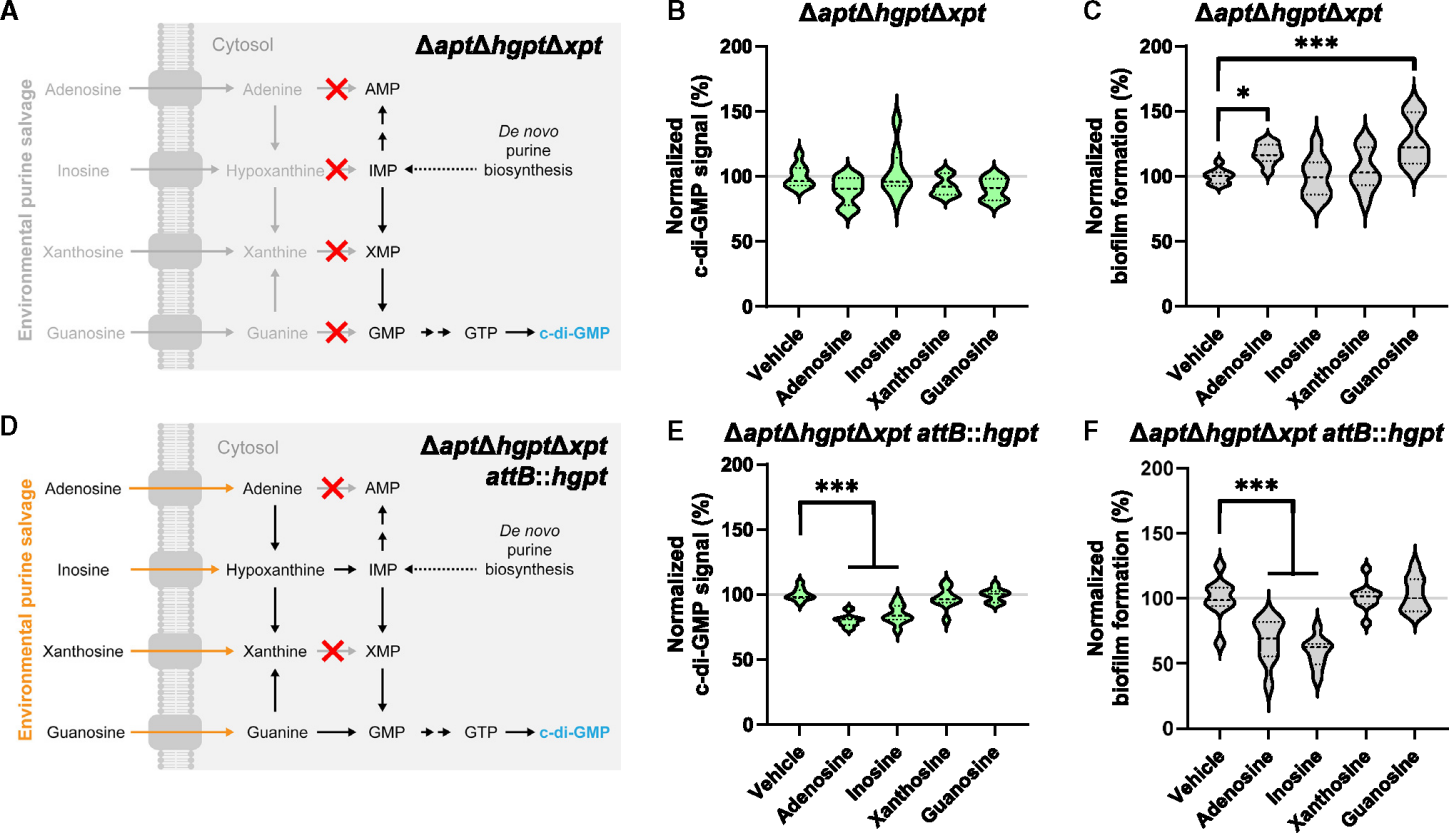
Adenosine- and inosine-dependent effects require purine salvage (A) Intracellular nucleotide pools are insulated from environmental purines in triple-salvage knockout Δ*apt*Δ*hgpt*Δ*xpt* background. (B) Violin plot of c-di-GMP signal from pCdrA::GFP(ASV) normalized to OD_600_ growth for Δ*apt*Δ*hgpt*Δ*xpt* exposed to vehicle or 100 μM indicated compounds after 8 h of exposure at 37°C with shaking. 3 wells per condition per experiment from 3 independent experiments were included (*n* = 9), barring outliers removed due to aberrant growth: *n* = 8 (adenosine, inosine, guanosine); *n* = 7 (xanthosine). (C) Violin plot of biofilm formation from safranin-stained biomass at OD_530_ normalized to OD_600_ growth for Δ*apt*Δ*hgpt*Δ*xpt* exposed to vehicle or 100 μM indicated compounds after 8 h of exposure. 3 wells per condition per experiment from 3 independent experiments were included (*n* = 9). (D) Intracellular nucleotide pools are no longer insulated from environmental purines in Δ*apt*Δ*hgpt*Δ*xpt attB::hgpt* background due to expression of *hgpt* from native promoter at the *attB* neutral site. (E) Violin plot of c-di-GMP signal from pCdrA::GFP(ASV) normalized to OD_600_ growth for Δ*apt*Δ*hgpt*Δ*xpt attB::hgpt* exposed to vehicle or 100 μM indicated compounds after 8 h of exposure at 37°C with shaking. 3 wells per condition per experiment from 3 independent experiments were included (*n* = 9), barring outliers removed due to aberrant growth: *n* = 7 (adenosine). (F) Violin plot of biofilm formation from safranin-stained biomass at OD_530_ normalized to OD_600_ growth for Δ*apt*Δ*hgpt*Δ*xpt attB::hgpt* exposed to vehicle or 100 μmM indicated compounds after 8 h of exposure. 3 wells per condition per experiment from 3 independent experiments were included (*n* = 9). Strains and compounds in (B) and (E) were tested simultaneously. Strains and compounds in (C) and (F) were tested simultaneously. *p ≤ 0.05, **p ≤ 0.01, and ***p ≤ 0.001. See also Figure S2.

We next suspected the purine salvage pathway to be responsible for c-di-GMP signaling. Accordingly, we created a salvage-deficient mutant, Δ*apt*Δ*hgpt*Δ*xpt*, which cannot catalyze the conversion of adenine to adenosine monophosphate (AMP) via adenine phosphoribosyltransferase (APT), hypoxanthine to inosine monophosphate (IMP) and guanine to GMP via hypoxanthine-guanine phosphoribosyltransferase (HGPT), or xanthine to xanthosine monophosphate (XMP) via xanthine phosphoribosyltransferase (XPT), respectively (Figure 2A). We deleted all three putative purine phosphoribosyltransferase genes to avoid cross-reactivity because the specificities of these enzymes are not well characterized in *P. aeruginosa*. We found that adenosine and inosine failed to decrease c-di-GMP and biofilm formation in the salvage-deficient mutant Δ*apt*Δ*hgpt*Δ*xpt* (Figures 2B and 2C). To confirm that the lack of effect is due to the intended genetic changes, we complemented *hgpt* genomically at the *attB* locus, a neutral chromosomal site, using the putative native *hgpt* promoter (Figure 2D). In this complemented *hgpt* strain, adenosine and inosine once again decreased c-di-GMP and biofilm formation (Figures 2E and 2F). These results indicate that the purine salvage pathway is required for purine-mediated c-di-GMP signaling.

### (p)ppGpp reinforces a nucleotide salvage hierarchy by preventing xanthosine- and guanosine-dependent effects

**Figure 3.**
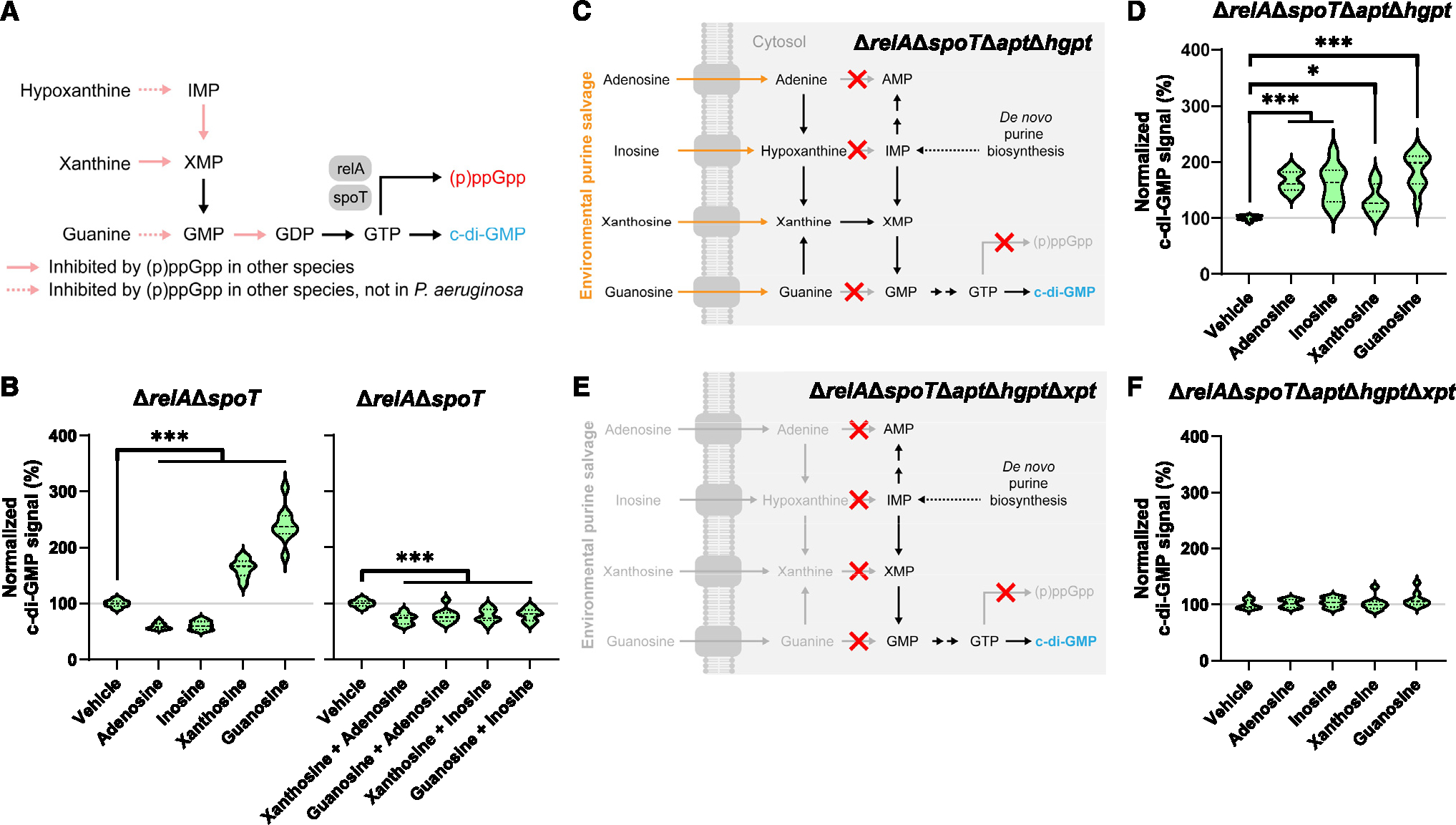
(p)ppGpp prevents xanthosine- and guanosine-dependent effects (A) (p)ppGpp inhibits GTP biosynthesis and salvage in other bacteria. (B) Violin plots of c-di-GMP signal from pCdrA::GFP(ASV) normalized to OD_600_ growth for Δ*relA*Δ*spoT* exposed to vehicle or 100 μM indicated compounds after 12 h of exposure at 37°C with shaking. 3 wells per condition per experiment from 3 independent experiments were included (*n* = 9), barring outliers removed due to aberrant growth: *n* = 8 (vehicle, xanthosine+adenosine, xanthosine+inosine); *n* = 7 (guanosine+adenosine). All compounds were tested simultaneously in the same experiments. (C) (p)ppGpp-mediated GTP homeostasis is absent in (p)ppGpp-null Δ*relA*Δ*spoT*Δ*apt*Δ*hgpt* background. (D) Violin plot of c-di-GMP signal from pCdrA::GFP(ASV) normalized to OD_600_ growth for Δ*relA*Δ*spoT*Δ*apt*Δ*hgpt* exposed to vehicle or 100 μM indicated compounds after 16 h of exposure at 37°C with shaking. 3 wells per condition per experiment from 3 independent experiments were included (*n* = 9), barring outliers removed due to aberrant growth: *n* = 7 (xanthosine). (E) (p)ppGpp-mediated GTP homeostasis is absent and intracellular nucleotide pools are insulated from environmental purines in (p)ppGpp-null Δ*relA*Δ*spoT*Δ*apt*Δ*hgpt*Δ*xpt* background. (F) Violin plot of c-di-GMP signal from pCdrA::GFP(ASV) normalized to OD_600_ growth for Δ*relA*Δ*spoT*Δ*apt*Δ*hgpt*Δ*xpt* exposed to vehicle or 100 μM indicated compounds after 16 h of exposure at 37°C with shaking. 3 wells per condition per experiment from 3 independent experiments were included (*n* = 9). Strains and compounds in (D) and (F) were tested simultaneously. *p ≤ 0.05, **p ≤ 0.01, and ***p ≤ 0.001. See also Figure S3.

We wondered why xanthosine and guanosine fail to affect c-di-GMP levels despite their ability to feed into the GTP pool. The second messenger (p)ppGpp is well known for its role in the stringent response to amino acid starvation. However, recent work has also revealed its involvement as a negative regulator for GTP biosynthesis in some bacteria (Figure 3A).^31–38^ Because GTP is the substrate for not only c-di-GMP synthesis but also (p)ppGpp synthesis, an increase in the GTP pool and the resulting increase in (p)ppGpp may subsequently inhibit further increases in GTP. We therefore hypothesized that (p)ppGpp may prevent xanthosine and guanosine from increasing c-di-GMP.

To test this hypothesis, we created a strain deficient in (p) ppGpp synthesis, Δ*relA*Δ*spoT*. We exposed this strain to different nucleosides and monitored c-di-GMP signal with pCdrA::GFP(ASV). Similar to MPAO1, adenosine and inosine decreased c-di-GMP signal by 40.7% (*n* = 9) and 38.8% (*n* = 9), respectively, in Δ*relA*Δ*spoT* (Figure 3B). Thus, (p)ppGpp does not appear to be involved in regulating the effects of adenosine and inosine on c-di-GMP levels. However, we found that xanthosine and guanosine produced large increases of 63.1% (*n* = 9) and 140.3% (*n* = 9), respectively, in c-di-GMP signal in the (p)ppGpp-null Δ*relA*Δ*spoT* (Figure 3B). These results suggest that (p)ppGpp—or a (p)ppGpp-dependent pathway—prevents xanthosine and guanosine from increasing c-di-GMP, reinforcing an apparent hierarchy of nucleotide salvage that favors c-di-GMP decrease.

### The nucleotide salvage hierarchy is maintained even in the absence of (p)ppGpp

We next investigated whether adenosine and inosine could block the large effects of xanthosine and guanosine on c-di-GMP in the absence of (p)ppGpp. To test this, we added equimolar pairwise mixtures of adenosine, inosine, xanthosine, and guanosine to Δ*relA*Δ*spoT*. Strikingly, each of these mixed purines decreased c-di-GMP to levels similar to adenosine and inosine alone (Figure 3B). Thus, even in the absence of (p)ppGpp, *P. aeruginosa* does not appear to integrate an average of available purines into its c-di-GMP response. Indeed, the presence of xanthosine and guanosine seems to be entirely ignored. Therefore, adenosine and inosine completely abolish the increase in c-di-GMP from xanthosine and guanosine due to the nucleotide salvage hierarchy.

To probe our understanding of the nucleotide salvage hierarchy, we reasoned that adenosine and inosine may be rewired to increase c-di-GMP if we prevented adenine and hypoxanthine from being converted to AMP and IMP, respectively. In a strain lacking *apt* and *hgpt*, only the xanthine-to-XMP purine phosphoribosyltransferase reaction should be possible. Since other nucleobases can interconvert to xanthine, it is plausible that all nucleosides may increase c-di-GMP in the (p)ppGpp-null background (Figure 3C). As expected, we found that adenosine, inosine, xanthosine, and guanosine all now produced large increases in c-di-GMP in Δ*relA*Δ*spoT*Δ*apt*Δ*hgpt* (Figure 3D). As before, these effects were dependent on purine salvage (Figures 3E and 3F). These targeted changes to purine metabolism that rewire the nucleotide salvage hierarchy demonstrate that adenosine and inosine mediate their effects on c-di-GMP levels via purine metabolism.

We also complemented *hgpt* into the *attB* locus of Δ*relA*Δ*spoT*Δ*apt*Δ*hgpt*Δ*xpt* to see if this would rescue the ability of adenosine and inosine to decrease c-di-GMP and the ability of guanosine to increase c-di-GMP (Figure S3J). Interestingly, while complementation of *hgpt* at the *attB* neutral chromosomal site rescued the ability of adenosine and inosine to decrease c-di-GMP, xanthosine and guanosine failed to affect c-di-GMP in the Δ*relA*Δ*spoT*Δ*apt*Δ*hgpt*Δ*xpt attB::hgpt* background (Figure S3L). It is not immediately clear why guanosine does not increase c-di-GMP in the Δ*relA*Δ*spoT*Δ*apt*Δ*hgpt*Δ*xpt attB::hgpt* background, but one possibility is that guanosine does not stimulate adequate expression of HGPT in this complement strain.

### The nucleotide salvage hierarchy is maintained even at the expense of growth

**Figure 4.**
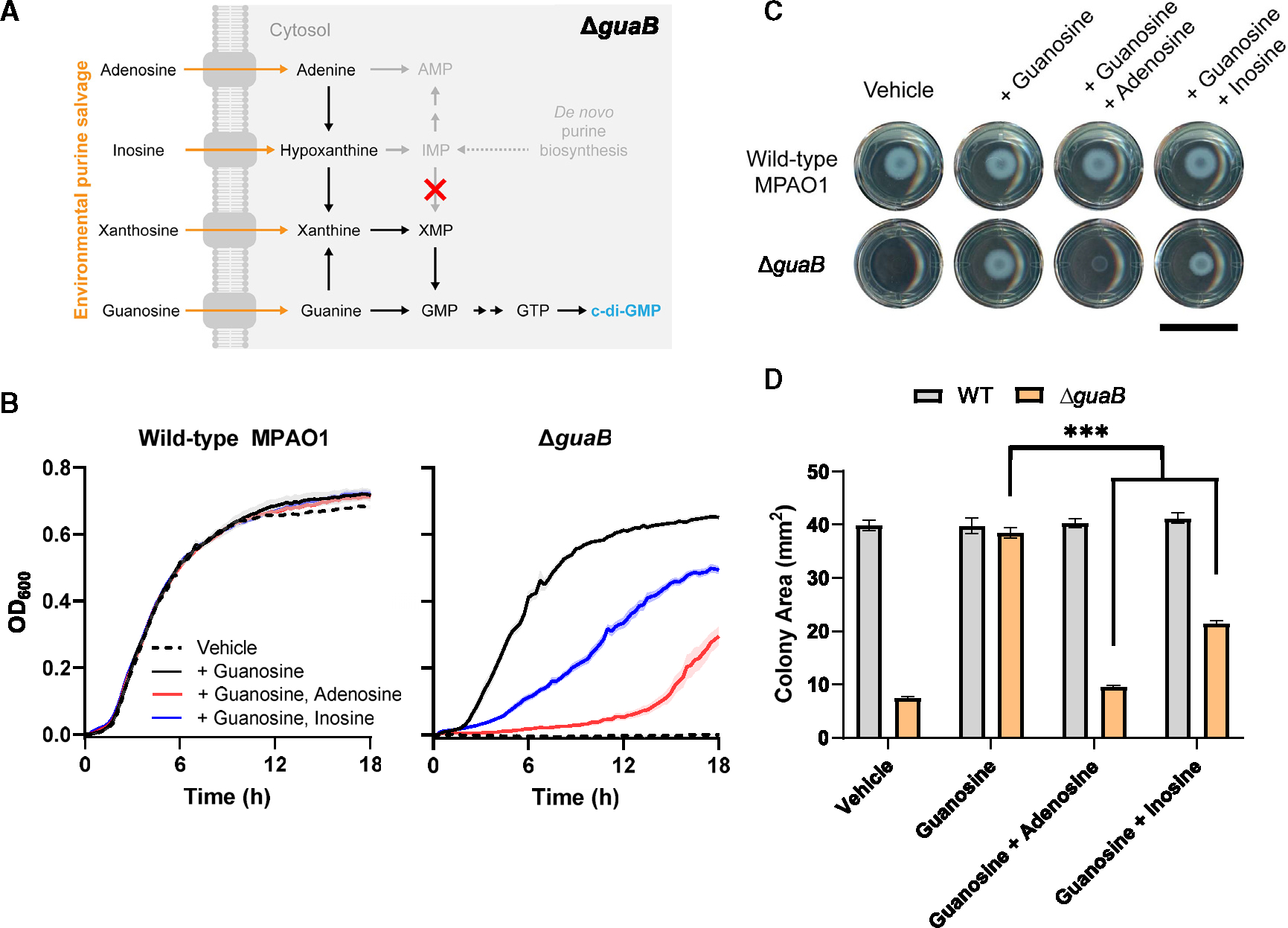
Guanine auxotroph reveals that adenosine and inosine block guanosine-dependent effects even at the expense of growth (A) *De novo* purine biosynthesis cannot convert IMP to XMP in Δ*guaB* background and consequently cannot synthesize GTP or grow unless supplemented with purines, preferentially guanosine. (B) Growth of MPAO1 and Δ*guaB* in M9 supplemented with vehicle or 500 μM of indicated compounds at 37°C with shaking. 3 wells per condition per experiment from 3 independent experiments (*n* = 9) were included, barring an outlier removed due to aberrant growth: *n* =8 (Δ*guaB* guanosine, inosine). Liquid growth curves for both strains and all compounds were tested simultaneously. Data represent mean ± SE. (C) Image of MPAO1 and Δ*guaB* growth on M9 agar pads supplemented with vehicle or 300 μM indicated compounds at 37°C after 24 h. 2 wells per condition per experiment from 4 independent experiments were included (*n* = 8). Agar-based growth for both strains and all compounds were tested simultaneously. Scale bar: 15 mm. (D) Bar plot of MPAO1 and Δ*guaB* colony size from (C) quantified using ImageJ. Data represent mean ± SE. ***p ≤ 0.001. See also Figure S4.

We wondered whether adenosine and inosine could block the salvage of xanthosine and guanosine even when vital to bacterial growth. To test this, we generated a strain lacking inosine monophosphate dehydrogenase, Δ*guaB*, which cannot convert IMP to XMP in *de novo* purine biosynthesis and therefore requires supplementation with purines to grow (Figures 4A, S4B–S4D, and S4F). We found that adenosine and inosine both inhibited growth of Δ*guaB* when included in liquid media containing guanosine (Figure 4B). These growth inhibitory effects appear to be dose dependent (Figure S4H). We also confirmed that these various nucleoside supplementations have no effect on the growth of wild-type MPAO1 (Figures 4B and S4H). We spotted cultures onto solid agar supplemented with guanosine or equimolar mixtures of guanosine and adenosine or inosine and found similar effects (Figures 4C and 4D). We observed the same effect with xanthosine supplementation, albeit to a lesser extent (Figures S4G–S4I and S4J). These results demonstrate that adenosine and inosine inhibit salvage of xanthosine and guanosine even when critical for growth, suggesting that the hierarchy of nucleotide salvage favoring c-di-GMP decrease may be hardwired in purine salvage and metabolism.

## DISCUSSION

Interest in bacterial second messengers has expanded in recent years,^39–48^ with c-di-GMP particularly noted for its ubiquity among bacteria and its central role in promoting the biofilm lifestyle.^49^ However, despite great progress on elucidating the genetic mechanisms of c-di-GMP homeostasis, comparatively few environmental cues that impact c-di-GMP levels have been identified. Our study reveals that micromolar levels of specific environmental purines can decrease c-di-GMP levels and biofilm formation in *P. aeruginosa* in a salvage-dependent manner. Salvage-dependent phenotypes have similarly been described for eukaryotes exposed to adenine or hypoxanthine, demonstrating that the effect of adenylate purines on *de novo* purine biosynthesis exists in a wide set of organisms.^50,51^ The requirement for an intact salvage pathway rules out direct action by purines themselves and suggests that the effect could be due to an increase in a salvage product that serves to disrupt c-di-GMP metabolism. In this context, while adenosine has been observed to affect biofilm formation of wild-type bacteria in a few cases,^52,53^ including once in *P. aeruginosa*,^54^ the studies have failed to make a mechanistic connection to purine metabolism and c-di-GMP signaling. Furthermore, the purine concentrations used in our study are 100-fold lower than those previously used for *P. aeruginosa*, which greatly expands the potential biomedical relevance of this phenomenon. We observed adenosine and inosine decrease biofilm formation of to not only MPAO1 but also *P. aeruginosa* strain PAK. While we did not observe adenosine or inosine to significantly decrease biofilm formation of PA14, PA14 is the *P. aeruginosa* strain with which an effect of adenosine on biofilm formation was previously observed. Collectively, our data and literature suggest that this biofilm response to purines is conserved across *P. aeruginosa* isolates, although the concentration or exposure time required for this effect may differ depending on which strain is used. Thus, our work reveals that adenosine and inosine can act as cues to decrease c-di-GMP and biofilm formation in *P. aeruginosa* through their effects on purine metabolism, significantly advancing our understanding of c-di-GMP metabolism for this model of biofilm formation and clinically relevant pathogens.

Our study reveals that the second messenger (p)ppGpp reinforces a hierarchy of nucleotide salvage favoring c-di-GMP decrease. Specifically, we showed that (p)ppGpp prevents xanthosine and guanosine from increasing c-di-GMP, that (p)ppGpp is not required for the inhibitory effect of adenosine and inosine, and that adenosine and inosine can both abolish the increase in c-di-GMP caused by xanthosine and guanosine. Together, these data suggest that adenosine and inosine repress a reaction late in *de novo* guanylate nucleotide biosynthesis necessary for guanylate nucleotide salvage, as hypothesized previously.^31^ Indeed, inhibition of Δ*guaB* growth by adenosine and inosine further demonstrates their capability to inhibit guanylate nucleotide salvage. Such growth inhibition was first described nearly 75 years ago and has been reported in a fungus and other bacteria, suggesting that this phenomenon may be widespread.^55–57^ In addition, enzymes in *de novo* guanylate nucleotide biosynthesis and salvage can be directly inhibited by (p)ppGpp to varying degrees.^31–37^ In *P. aeruginosa*, both HGPT and guanosine monophosphate kinase (GMK) are thought to be resistant to (p)ppGpp,^35^ which leaves the mechanism(s) by which (p)ppGpp prevents salvage of xanthosine and guanosine unclear. Further study will be required to elucidate how (p)ppGpp maintains GTP homeostasis in *P. aeruginosa*, especially in conditions in which xanthosine or guanosine are present in the environment.

It is commonly assumed that intracellular nucleotide pools remain in excess and are therefore irrelevant for nucleotide-derived second messenger signaling. Our work revises this assumption by showing that environmental purines can influence c-di-GMP levels, both negatively and positively, by disrupting c-di-GMP precursor metabolism. This paradigm of upstream metabolic control of c-di-GMP signaling may lead to a mechanistic explanation for the ubiquitous observation of mutual antagonism between cyclic AMP and c-di-GMP across bacterial species. In the context of infection, while the concentration of purines in extracellular fluids is generally low, the intracellular concentration of purines in human cells is more than sufficient to trigger the effects we observe.^58^ Therefore, such c-di-GMP signaling may be triggered by nearby damaged or diseased tissues,^59–62^ bacterial invasion of host cells,^63,64^ or other host events in which environmental purines become elevated.^65^ Release of adenosine and adenosine triphosphate (ATP) as used in receptor-mediated purinergic signaling also represents a potential source of purines, although the concentration of these compounds may be too low to influence c-di-GMP levels.^66^ Thus, in contrast to the common assumption in the field, alterations in intracellular nucleotide pools may influence multiple phenotypes associated with these nucleotide-derived second messengers including virulence, biofilm formation, and antimicrobial resistance.^67^

### Limitations of the study

This study explored the effect of environmental purines on intracellular c-di-GMP and biofilm formation in *P. aeruginosa* MPAO1. Although this response may be conserved across organisms, we did not determine the extent to which this purine response exists in a comprehensive set of bacterial strains or species. Additionally, while our reporter experiments provide some temporal insight regarding the c-di-GMP response to purines, the temporal dynamic of c-di-GMP itself is largely lost due to delays inherent to biological processes when using a transcriptional fluorescent reporter, such as transcription, translation, fluorophore maturation, and protein turnover, particularly when c-di-GMP levels decrease. Our work demonstrated that the c-di-GMP and biofilm response to adenosine and inosine requires purine salvage enzymes. However, the precise target that is affected by purines remains to be determined. If the target is an enzyme in *de novo* purine biosynthesis as we hypothesize, then generating a mutant that is insensitive to these purines due to a mutation in the target enzyme may be challenging because these enzymes are strictly required for survival and may not tolerate significant alteration. Finally, while we verified that the purine response occurs even when xanthosine or guanosine is provided with adenosine and inosine in M9 media, we do not know whether this response occurs in more complex environments, such as those found in human infection.

## STAR★METHODS

### RESOURCE AVAILABILITY

#### Lead contact

Further information and requests for resources and reagents should be directed to and will be fulfilled by the lead contact, Arthur Prindle (arthur.prindle@northwestern.edu).

#### Materials availability

Bacterial strains and plasmids generated in this study are available upon request.

#### Data and code availability

- All data reported in the paper are available from the lead contact upon request.
- This paper does not report original code.
- Any additional information required to reanalyze the data reported in this paper is available from the lead contact upon request.

### EXPERIMENTAL MODEL AND STUDY PARTICIPANT DETAILS

#### Bacterial strains and growth conditions

*P. aeruginosa* MPAO1 was obtained from the University of Washington.^68^ Bacteria were frozen in 50% glycerol-50% Luria-Bertani (LB) media (Fisher Bioreagents) and stored at −80°C, except guanine auxotroph strains which were frozen in 50% glycerol-50% 500 μM guanosine (TCI) M9 media. M9 media contained 47.7 mM Na_2_HPO_4_ (Sigma-Aldrich), 21.7 mM KH_2_PO_4_ (Sigma-Aldrich), 18.7 mM NH_4_Cl (Fisher Chemical), 8.6 mM NaCl (Sigma-Aldrich), 0.5% acid casein peptone (Fisher Bioreagents), 0.2% glucose (Sigma-Aldrich), and 1 mM MgSO_4_ (Sigma-Aldrich). Solid media was prepared by adding 1.5 g/L agar (Fisher Bioreagents)) to the liquid media before autoclaving. *Escherichia coli* and *Pseudomonas aeruginosa* were routinely grown on LB agar at 37°C overnight and single colonies were used to inoculate LB media for growth at 37°C overnight with shaking at 250 rpm unless otherwise stated. Δ*guaB* was grown on M9 agar equivalents to LB agar used by non-auxotroph MPAO1 strains. For use in experiments, *P. aeruginosa* was grown in M9 media. When appropriate, 50 μg/mL (*E. coli*) or 250 μg/mL (*P. aeruginosa*) carbenicillin (Sigma-Aldrich), 15 μg/mL (*E. coli*) or 50 μg/mL (*P. aeruginosa*) gentamicin (TCI), and/or 5 μg/mL (*P. aeruginosa*) irgasan (Sigma-Aldrich) was added to media for selection. 7.5% sucrose (Sigma-Aldrich) was added to no-salt LB agar – 10 g/L tryptone (Fisher Bioreagents) and 5 g/L yeast extract (Fisher Bioreagents) – for sucrose counterselection. Single colonies of *P. aeruginosa* were inoculated into M9 media for growth at 37°C overnight for c-di-GMP reporter and biofilm staining experiments.

### METHOD DETAILS

#### Generation of knockout strains

Allelic exchange was used to generate clean-deletion knockouts of *P. aeruginosa* MPAO1. Briefly, regions upstream and downstream of genes of interest were amplified from the MPAO1 genome by PCR including Gibson overhangs using Phusion Green Hot Start II High-Fidelity PCR Master Mix (New England BioLabs). pEXG2 vector was digested with HinDIII-HF (New England BioLabs).^69^ Upstream and downstream regions were combined in three-part Gibson assembly with cut pEXG2 using Gibson Assembly Master Mix (New England BioLabs). NEB 5-alpha Competent *E. coli* (New England BioLabs) was chemically transformed with constructs and insert presence was detected by PCR and verified by Sanger sequencing. *E. coli* S17–1^70^ was chemically transformed with verified constructs. Constructs were then mated into *P. aeruginosa* by conjugation. Sucrose counterselection was used to resolve merodiploids. Knockouts were verified by amplifying regions of interest by PCR and sequencing with Sanger sequencing and/or Nanopore sequencing. For generation of Δ*guaB* strain, 150 μM guanosine was included in plates used for merodiploid selection and 3000 μM guanosine was included in sucrose plates used for merodiploid resolution.

#### Generation of complement strain

*hgpt* was complemented genomically at the *attB* locus using the pminiCTX system.^71^ Briefly, *PA4645* (*hgpt*) was amplified from the MPAO1 genome by PCR including Gibson overhangs and combined in two-part Gibson assembly with HindIII-digested pminiCTX-1. A 168 basepair region upstream of *PA4645* was included in this amplification based on Sapphire promoter prediction software.^72^ NEB 5-alpha Competent *E. coli* was chemically transformed with pminiCTX-1-HGPT construct and insert presence was detected by PCR and verified by Sanger sequencing. S17–1 was chemically transformed with verified construct, which was then mated into Δ*apt*Δ*hgpt*Δ*xpt* and Δ*relA*Δ*spoT*Δ*apt*Δ*hgpt*Δ*xpt* by conjugation. Conjugants were selected for with gentamicin and irgasan. pFLP2^73^ was then mated into these strains by Sm10^70^ and conjugants were selected for with carbenicillin and irgasan. Expression of flp recombinase to remove undesired integrated pminiCTX remnants was ensured by inoculating colonies into LB media containing carbenicillin and irgasan and growing overnight at 37°C before using sucrose counterselection to remove pFLP2. Complements were verified by amplifying *attB* site by PCR and sequencing with Sanger sequencing and/or Nanopore sequencing.

#### Quantification of c-di-GMP signal

40 μL of 500 μM stock compounds of adenosine (TCI), inosine (TCI), xanthosine (TCI), guanosine (TCI), adenine (Alfa Aesar), and hypoxanthine (Thermo Scientific) dissolved in water or water (vehicle) were added to 160 μL OD_600_ ~1 MPAO1 or relevant genetic knockout strain containing pCdrA::GFP(ASV) grown on M9. pCdrA-gfp(ASV)C was a gift from Tim Tolker-Nielsen (Addgene plasmid # 111615; http://n2t.net/addgene:111615; RRID:Addgene_111615). Final concentration for all compounds was 100 μM. Black 96 well plates with clear bottoms (Nunc, Thermo Scientific) were covered with gas-permeable Breathe-Easy film (USA Scientific) and shaken at 37°C in Tecan Infinite MPlex plate reader with an absorbance measurement at 600 nm and bottom fluorescence measurement taken every 15 min for at least 16 h. Excitation wavelength was 485 ± 9 nm while emission wavelength was 515 ± 20 nm. Gain was set to 180. MPAO1 or relevant genetic knockout strain not containing pCdrA::GFP(ASV) was grown in parallel and mean fluorescence of this strain was subtracted from GFP signal to account for autofluorescence. Mean OD_600_ values from media-only wells were subtracted as background. GFP signal was divided by OD_600_ measurements to normalize for growth. GFP/OD_600_ signal for all compounds was then normalized relative to that of vehicle. Therefore, compounds that affect GFP/OD_600_ signal the same as vehicle should match the vehicle line at 100% normalized c-di-GMP signal. For violin plots, the data for each replicate consists of the mean GFP/OD_600_ signal for the period from half an hour before to half an hour after the stated time point. Plots were generated with GraphPad Prism. Repeated measures one-way ANOVA with Dunnett’s multiple comparison test comparing to vehicle was used to determine statistical significance.

#### Quantification of biofilm formation

This safranin biofilm assay was based on work by Ommen et al.^74^ 2 μL overnight culture of MPAO1 or relevant genetic knockout strain grown in M9 was inoculated into 198 μL M9 supplemented with 100 μM compounds or vehicle and grown statically in clear 96-well plates (Nunc, Thermo Scientific) at 37°C. 50 mM arginine (Dot Scientific) was included in media for the experiment comparing biofilm formation of MPAO1, Δ*xdhA*, Δ*apt*Δ*hgpt*Δ*xpt*, and Δ*apt*Δ*hgpt*Δ*xpt attB::hgpt*. After 8 h, an OD_600_ measurement was taken in Synergy *Neo*2 plate reader (BioTek). Liquid was removed and wells were allowed to air dry for at least 30 min. Wells were stained with 200 μL of 0.42% safranin (Alfa Aesar) for 10 min and then washed thrice with water to remove unbound dye and unattached biomass. Wells were then allowed to air dry for 30 min. Bound safranin was solubilized with 200 μL of 30% acetic acid (Fisher Scientific) and OD_530_ was measured in Synergy *Neo*2 plate reader after an additional 30 min. Mean OD_600_ and OD_530_ values from media-only wells were subtracted as background. OD_530_ was divided by OD_600_ to normalize for growth. Plots were generated with GraphPad Prism. Repeated measures one-way ANOVA with Dunnett’s multiple comparison test comparing to vehicle was used to determine statistical significance.

#### Quantification of growth in liquid media

*P. aeruginosa* was grown overnight in M9 supplemented with 500 μM guanosine. MPAO1 and Δ*guaB* were pelleted via centrifugation and resuspended twice in M9 to remove guanosine. 2 μL of these washed cultures was then inoculated into 198 μL of M9 supplemented with indicated compounds in clear 96-well plates, wells were covered with Breathe-Easy film, and plates were shaken at 37°C in Synergy *Neo*2 plate reader with an absorbance measurement at 600 nm taken every 15 min for at least 18 h. Initial OD_600_ value for each well was subtracted from all time points of that well as background. Plots were generated with GraphPad Prism.

#### Imaging and quantification of growth on solid agar

*P. aeruginosa* was grown overnight in M9 supplemented with 500 μM guanosine. MPAO1 and Δ*guaB* were pelleted via centrifugation and resuspended twice in M9 to remove guanosine. 2 μL of these washed cultures was then spotted onto M9 agar pads supplemented with indicated compounds in clear Costar 24-well plates (Corning) and wells were covered with Breathe-Easy film. Bacteria were grown statically at 37°C. After 24 h, plates were imaged using an Epson Perfection V550 Photo scanner. Area of bacterial growth for each agar pad was individually calculated with ImageJ.^75^ Plots were generated with GraphPad Prism. Repeated measures one-way ANOVA with Dunnett’s multiple comparison test comparing to vehicle was used to determine statistical significance.

### QUANTIFICATION AND STATISTICAL ANALYSIS

Analyses were performed using GraphPad Prism v10..2..2. Data are represented with violin plots for c-di-GMP signal and biofilm formation experiments. For growth curve experiments, absorbance data are represented with mean ± standard error (SE) for each time point. For quantification of growth on solid agar, colony area size data are represented with mean ± standard error (SE). For statistical analysis throughout this work, repeated measures one-way ANOVA with Dunnett’s multiple comparison test was used to determine statistical significance. *p ≤ 0.05; **p ≤ 0.01; ***p ≤ 0.001.

## Supplementary Material

1

## Figures and Tables

**KEY RESOURCES TABLE:** 

REAGENT or RESOURCE	SOURCE	IDENTIFIER

Bacterial and virus strains		

NEB 5-alpha Competent E. coli	New England Biolabs	N/A
S17–1 *λpir*	Simon et al. (1983)^70^	N/A
SM10 *λpir*	Simon etal. (1983)^70^	N/A
MPAO1	Jacobs et al. (2003)^68^	N/A
MPAO1 *ΔxdhA*	This study	N/A
MPAO1 *ΔaptΔhgptΔxpt*	This study	N/A
MPAO1 *ΔaptΔhgptΔxpt attB::hgpt*	This study	N/A
MPAO1 *ΔrelAΔspoT*	This study	N/A
MPAO1 *ΔrelAΔspoTΔaptΔhgpt*	This study	N/A
MPAO1 *ΔrelAΔspoTΔaptΔhgptΔxpt*	This study	N/A
MPAO1 *ΔrelAΔspoTΔaptΔhgptΔxpt attB::hgpt*	This study	N/A
MPAO1 *ΔguaB*	This study	N/A
PAK	Takeya and Amako (1966)^29^	N/A
PA14	Schroth et al. (2018)^30^	N/A

Chemicals, peptides, and recombinant proteins		

Acetic Acid	Fisher Scientific	A38–212
Acid Casein Peptone	Fisher Bioreagents	BP1424–100
Adenine	Alfa Aesar	A14906
Adenosine	TCI	A0152
Agar	Fisher Bioreagents	BP1423–500
Ammonium Chloride	Fisher Chemical	A661–500
Arginine	Dot Scientific	DSA50400–100
Breathe-Easy Film	USA Scientific	9123–6100
Carbenicillin	Sigma-Aldrich	C9231–1G
Costar 24-well Clear TC-treated Multiple Well Plates	Corning	3526
Dextrose (Glucose)	Sigma-Aldrich	D9434–500G
Gentamicin	TCI	G0383
Gibson Assembly Master Mix	New England BioLabs	M5510AA
Glycerol	Sigma-Aldrich	G5516–1L
Guanosine	TCI	G0171
HinDIII-HF	New England BioLabs	R3104S
Hypoxanthine	Thermo Scientific	122010050
Inosine	TCI	I0037
Irgasan	Sigma-Aldrich	72779–5G-F
Luria-Bertani Powder	Fisher Bioreagents	BP1427–2
Magnesium Sulfate Heptahydrate	Sigma-Aldrich	BP213–1
Nunc MicroWell 96-Well Microplates	Thermo Scientific	167008
Nunc MicroWell 96-Well Optical-Bottom Plates with Black Polymer Base	Thermo Scientific	165305
Phusion Green Hot Start II High-Fidelity PCR Master Mix	New England BioLabs	F566L
Potassium Phosphate Monobasic	Sigma-Aldrich	795488–500G
Safranin O	Alfa Aesar	B21674.18
Sodium Chloride	Sigma-Aldrich	S9888–10KG
Sodium Phosphate Dibasic	Sigma-Aldrich	S7907–500G
Sucrose	Sigma-Aldrich	S0389–1KG
Tryptone	Fisher Bioreagents	BP1421–2
Xanthosine	TCI	X0008
Yeast Extract	Fisher Bioreagents	BP1422–500

Oligonucleotides		

See Table S1		

Recombinant DNA		

pCdrA::GFP(ASV)	Rybtke et al. (2012)^28^	N/A
pEXG2	Rietsch et al. (2005)^69^	N/A
pEXG2-xdhA	This study	N/A
pEXG2-APT	This study	N/A
pEXG2-HGPT	This study	N/A
pEXG2-XPT	This study	N/A
pEXG2-guaB	This study	N/A
pEXG2-relA	This study	N/A
pEXG2-spoT	This study	N/A
pminiCTX-1 (GmR derivative)	Hoang et al. (2000)^71^	N/A
pminiCTX-1-HGPT	This study	N/A
pFLP2	Hoang et al. (1998)^73^	N/A

Software and algorithms		

GraphPad Prism v10.2.2	GraphPad	www.graphpad.com
ImageJ	Available from NIH	https://imagej.net

## Highlights

- Adenosine and inosine decrease c-di-GMP and biofilm formation
- When (p)ppGpp is not present, xanthosine and guanosine increase c-di-GMP
- Adenosine and inosine block the effects of xanthosine and guanosine
- Hierarchy in purine salvage thus favors c-di-GMP decrease

## References

[1] Flemming HC, and Wuertz S (2019). Bacteria and archaea on Earth and their abundance in biofilms. Nat. Rev. Microbiol. 17, 247–260. 10.1038/s41579-019-0158-9. 30760902

[2] Schultz MP, Bendick JA, Holm ER, and Hertel WM (2011). Economic impact of biofouling on a naval surface ship. Biofouling 27, 87–98. 10.1080/08927014.2010.542809. 21161774

[3] Khatoon Z, McTiernasn CD, Suuronen EJ, Mah TF, and Alarcon EI (2018). Bacterial biofilm formation on implantable devices and approaches to its treatment and prevention. Heliyon 4, e01067. 10.1016/j.heliyon.2018.e01067. 30619958 PMC6312881

[4] Sharma D, Misba L, and Khan AU (2019). Antibiotics versus biofilm: an emerging battleground in microbial communities. Antimicrob. Resist. Infect. Control 8, 76. 10.1186/s13756-019-0533-3. 31131107 PMC6524306

[5] Malone M, Bjarnsholt T, McBain AJ, James GA, Stoodley P, Leaper D, Tachi M, Schultz G, Swanson T, and Wolcott RD (2017). The prevalence of biofilms in chronic wounds: a systematic review and meta-analysis of published data. J. Wound Care 26, 20–25. 10.12968/jowc.2017.26.1.20. 28103163

[6] Antimicrobial Resistance Collaborators (2022). Global burden of bacterial antimicrobial resistance in 2019: a systematic analysis. Lancet 399, 629–655. 10.1016/S0140-6736(21)02724-0. 35065702 PMC8841637

[7] Valentini M, and Filloux A (2016). Biofilms and Cyclic di-GMP (c-di-GMP) Signaling: Lessons from Pseudomonas aeruginosa and Other Bacteria. J. Biol. Chem. 291, 12547–12555. 10.1074/jbc.R115.711507. 27129226 PMC4933438

[8] Ryjenkov DA, Tarutina M, Moskvin OV, and Gomelsky M (2005). Cyclic diguanylate is a ubiquitous signaling molecule in bacteria: insights into biochemistry of the GGDEF protein domain. J. Bacteriol. 187, 1792–1798. 10.1128/JB.187.5.1792-1798.2005. 15716451 PMC1064016

[9] Schmidt AJ, Ryjenkov DA, and Gomelsky M (2005). The ubiquitous protein domain EAL is a cyclic diguanylate-specific phosphodiesterase: enzymatically active and inactive EAL domains. J. Bacteriol. 187, 4774–4781. 10.1128/JB.187.14.4774-4781.2005. 15995192 PMC1169503

[10] Stelitano V, Giardina G, Paiardini A, Castiglione N, Cutruzzolà F, and Rinaldo S (2013). C-di-GMP hydrolysis by Pseudomonas aeruginosa HD-GYP phosphodiesterases: analysis of the reaction mechanism and novel roles for pGpG. PLoS One 8, e74920. 10.1371/journal.pone.0074920. 24066157 PMC3774798

[11] Cohen D, Mechold U, Nevenzal H, Yarmiyhu Y, Randall TE, Bay DC, Rich JD, Parsek MR, Kaever V, Harrison JJ, and Banin E (2015). Oligoribonuclease is a central feature of cyclic diguanylate signaling in Pseudomonas aeruginosa. Proc. Natl. Acad. Sci. USA 112, 11359–11364. 10.1073/pnas.1421450112. 26305928 PMC4568660

[12] Orr MW, Donaldson GP, Severin GB, Wang J, Sintim HO, Waters CM, and Lee VT (2015). Oligoribonuclease is the primary degradative enzyme for pGpG in Pseudomonas aeruginosa that is required for cyclic-di-GMP turnover. Proc. Natl. Acad. Sci. USA 112, E5048–E5057. 10.1073/pnas.1507245112. 26305945 PMC4568665

[13] Seshasayee ASN, Fraser GM, and Luscombe NM (2010). Comparative genomics of cyclic-di-GMP signalling in bacteria: post-translational regulation and catalytic activity. Nucleic Acids Res. 38, 5970–5981. 10.1093/nar/gkq382. 20483912 PMC2952852

[14] O’Neal L, Baraquet C, Suo Z, Dreifus JE, Peng Y, Raivio TL, Wozniak DJ, Harwood CS, and Parsek MR (2022). The Wsp system of Pseudomonas aeruginosa links surface sensing and cell envelope stress. Proc. Natl. Acad. Sci. USA 119, e2117633119. 10.1073/pnas.2117633119. 35476526 PMC9170161

[15] Roy AB, Petrova OE, and Sauer K (2012). The phosphodiesterase DipA (PA5017) is essential for Pseudomonas aeruginosa biofilm dispersion. J. Bacteriol. 194, 2904–2915. 10.1128/JB.05346-11. 22493016 PMC3370607

[16] Monds RD, Newell PD, Wagner JC, Schwartzman JA, Lu W, Rabinowitz JD, and O’Toole GA (2010). Di-adenosine tetraphosphate (Ap4A) metabolism impacts biofilm formation by Pseudomonas fluorescens via modulation of c-di-GMP-dependent pathways. J. Bacteriol. 192, 3011–3023. 10.1128/JB.01571-09. 20154123 PMC2901679

[17] Yoshioka S, and Newell PD (2016). Disruption of de novo purine biosynthesis in Pseudomonas fluorescens Pf0–1 leads to reduced biofilm formation and a reduction in cell size of surface-attached but not planktonic cells. PeerJ 4, e1543. 10.7717/peerj.1543. 26788425 PMC4715448

[18] Gélinas M, Museau L, Milot A, and Beauregard PB (2021). The de novo Purine Biosynthesis Pathway Is the Only Commonly Regulated Cellular Pathway during Biofilm Formation in TSB-Based Medium in Staphylococcus aureus and Enterococcus faecalis. Microbiol. Spectr. 9, e0080421. 10.1128/Spectrum.00804-21. 34935415 PMC8693917

[19] Shaffer CL, Zhang EW, Dudley AG, Dixon BREA, Guckes KR, Breland EJ, Floyd KA, Casella DP, Algood HMS, Clayton DB, and Hadjifrangiskou M (2017). Purine Biosynthesis Metabolically Constrains Intracellular Survival of Uropathogenic Escherichia coli. Infect. Immun. 85, e00471–16. 10.1128/IAI.00471-16. PMC5203662 27795353

[20] Antoniani D, Rossi E, Rinaldo S, Bocci P, Lolicato M, Paiardini A, Raffaelli N, Cutruzzolà F, and Landini P (2013). The immunosuppressive drug azathioprine inhibits biosynthesis of the bacterial signal molecule cyclic-di-GMP by interfering with intracellular nucleotide pool availability. Appl. Microbiol. Biotechnol. 97, 7325–7336. 10.1007/s00253-013-4875-0. 23584245

[21] Al Mahmud H, Baishya J, and Wakeman CA (2021). Interspecies Metabolic Complementation in Cystic Fibrosis Pathogens via Purine Exchange. Pathogens 10, 146. 10.3390/pathogens10020146. 33535659 PMC7912780

[22] Kilstrup M, Hammer K, Ruhdal Jensen P, and Martinussen J (2005). Nucleotide metabolism and its control in lactic acid bacteria. FEMS Microbiol. Rev. 29, 555–590. 10.1016/j.femsre.2005.04.006. 15935511

[23] Bagnara AS, and Finch LR (1974). The effects of bases and nucleosides on the intracellular contents of nucleotides and 5-phosphoribosyl 1-pyrophosphate in Escherichia coli. Eur. J. Biochem. 41, 421–430. 10.1111/j.1432-1033.1974.tb03283.x. 4361644

[24] Hosono R, and Kuno S (1974). Mechanism of inhibition of bacterial growth by adenine. J. Biochem. 75, 215–220. 10.1093/oxfordjournals.jbchem.a130388. 4209780

[25] Petersen C (1999). Inhibition of cellular growth by increased guanine nucleotide pools. Characterization of an Escherichia coli mutant with a guanosine kinase that is insensitive to feedback inhibition by GTP. J. Biol. Chem. 274, 5348–5356. 10.1074/jbc.274.9.5348. 10026143

[26] Entezampour M (1988). Quantitation of Endogenous Nucleotide Pools in *Pseudomonas aeruginosa*. Master of Science (University of North Texas).

[27] Levine RA, and Taylor MW (1982). Mechanism of adenine toxicity in Escherichia coli. J. Bacteriol. 149, 923–930. 10.1128/jb.149.3.923-930.1982. 6801015 PMC216479

[28] Rybtke MT, Borlee BR, Murakami K, Irie Y, Hentzer M, Nielsen TE, Givskov M, Parsek MR, and Tolker-Nielsen T (2012). Fluorescence-based reporter for gauging cyclic di-GMP levels in Pseudomonas aeruginosa. Appl. Environ. Microbiol. 78, 5060–5069. 10.1128/AEM.00414-12. 22582064 PMC3416407

[29] Takeya K, and Amako K (1966). A rod-shaped Pseudomonas phage. Virology 28, 163–165. 10.1016/0042-6822(66)90317-5. 4955194

[30] Schroth MN, Cho JJ, Green SK, Kominos SD, and Microbiology Society P (2018). Epidemiology of Pseudomonas aeruginosa in agricultural areas. J. Med. Microbiol. 67, 1191–1201. 10.1099/jmm.0.000758. 30067169

[31] Bittner AN, Kriel A, and Wang JD (2014). Lowering GTP level increases survival of amino acid starvation but slows growth rate for Bacillus subtilis cells lacking (p)ppGpp. J. Bacteriol. 196, 2067–2076. 10.1128/JB.01471-14. 24682323 PMC4010990

[32] Anderson BW, Hao A, Satyshur KA, Keck JL, and Wang JD (2020). Molecular Mechanism of Regulation of the Purine Salvage Enzyme XPRT by the Alarmones pppGpp, ppGpp, and pGpp. J. Mol. Biol. 432, 4108–4126. 10.1016/j.jmb.2020.05.013. 32446804 PMC7323586

[33] Gaca AO, Kajfasz JK, Miller JH, Liu K, Wang JD, Abranches J, and Lemos JA (2013). Basal levels of (p)ppGpp in Enterococcus faecalis: the magic beyond the stringent response. mBio 4, e00646–e00613. 10.1128/mBio.00646-13. 24065631 PMC3781836

[34] Gallant J, Irr J, and Cashel M (1971). The mechanism of amino acid control of guanylate and adenylate biosynthesis. J. Biol. Chem. 246, 5812–5816. 4938039

[35] Liu K, Myers AR, Pisithkul T, Claas KR, Satyshur KA, Amador-Noguez D, Keck JL, and Wang JD (2015). Molecular mechanism and evolution of guanylate kinase regulation by (p)ppGpp. Mol. Cell. 57, 735–749. 10.1016/j.molcel.2014.12.037. 25661490 PMC4336630

[36] Nomura Y, Izumi A, Fukunaga Y, Kusumi K, Iba K, Watanabe S, Nakahira Y, Weber APM, Nozawa A, and Tozawa Y (2014). Diversity in guanosine 3’,5’-bisdiphosphate (ppGpp) sensitivity among guanylate kinases of bacteria and plants. J. Biol. Chem. 289, 15631–15641. 10.1074/jbc.M113.534768. 24722991 PMC4140918

[37] Wang B, Dai P, Ding D, Del Rosario A, Grant RA, Pentelute BL, and Laub MT (2019). Affinity-based capture and identification of protein effectors of the growth regulator ppGpp. Nat. Chem. Biol. 15, 141–150. 10.1038/s41589-018-0183-4. 30559427 PMC6366861

[38] Kriel A, Bittner AN, Kim SH, Liu K, Tehranchi AK, Zou WY, Rendon S, Chen R, Tu BP, and Wang JD (2012). Direct regulation of GTP homeostasis by (p)ppGpp: a critical component of viability and stress resistance. Mol. Cell. 48, 231–241. 10.1016/j.molcel.2012.08.009. 22981860 PMC3483369

[39] Yoon SH, and Waters CM (2021). The ever-expanding world of bacterial cyclic oligonucleotide second messengers. Curr. Opin. Microbiol. 60, 96–103. 10.1016/j.mib.2021.01.017. 33640793 PMC8026173

[40] McDonough KA, and Rodriguez A (2011). The myriad roles of cyclic AMP in microbial pathogens: from signal to sword. Nat. Rev. Microbiol. 10, 27–38. 10.1038/nrmicro2688. 22080930 PMC3785115

[41] Steinchen W, Zegarra V, and Bange G (2020). (p)ppGpp: Magic Modulators of Bacterial Physiology and Metabolism. Front. Microbiol. 11, 2072. 10.3389/fmicb.2020.02072. 33013756 PMC7504894

[42] Stülke J, and Krüger L (2020). Cyclic di-AMP Signaling in Bacteria. Annu. Rev. Microbiol. 74, 159–179. 10.1146/annurev-micro-020518-115943. 32603625

[43] Giammarinaro PI, Young MKM, Steinchen W, Mais CN, Hochberg G, Yang J, Stevenson DM, Amador-Noguez D, Paulus A, Wang JD, and Bange G (2022). Diadenosine tetraphosphate regulates biosynthesis of GTP in Bacillus subtilis. Nat. Microbiol. 7, 1442–1452. 10.1038/s41564-022-01193-x. 35953658 PMC10439310

[44] Whiteley AT, Eaglesham JB, de Oliveira Mann CC, Morehouse BR, Lowey B, Nieminen EA, Danilchanka O, King DS, Lee ASY, Mekalanos JJ, and Kranzusch PJ (2019). Bacterial cGAS-like enzymes synthesize diverse nucleotide signals. Nature 567, 194–199. 10.1038/s41586-019-0953-5. 30787435 PMC6544370

[45] Millman A, Melamed S, Amitai G, and Sorek R (2020). Diversity and classification of cyclic-oligonucleotide-based anti-phage signalling systems. Nat. Microbiol. 5, 1608–1615. 10.1038/s41564-020-0777-y. 32839535 PMC7610970

[46] Hobbs SJ, Wein T, Lu A, Morehouse BR, Schnabel J, Leavitt A, Yirmiya E, Sorek R, and Kranzusch PJ (2022). Phage anti-CBASS and anti-Pycsar nucleases subvert bacterial immunity. Nature 605, 522–526. 10.1038/s41586-022-04716-y. 35395152 PMC9117128

[47] Luo Y, Zhao K, Baker AE, Kuchma SL, Coggan KA, Wolfgang MC, Wong GCL, and O’Toole GA (2015). A hierarchical cascade of second messengers regulates Pseudomonas aeruginosa surface behaviors. mBio 6, e02456–14. 10.1128/mBio.02456-14. 25626906 PMC4324313

[48] Maronde E (2021). Cyclic Nucleotide (cNMP) Analogues: Past, Present and Future. Int. J. Mol. Sci. 22, 12879. 10.3390/ijms222312879. 34884683 PMC8657615

[49] Jenal U, Reinders A, and Lori C (2017). Cyclic di-GMP: second messenger extraordinaire. Nat. Rev. Microbiol. 15, 271–284. 10.1038/nrmicro.2016.190. 28163311

[50] Dienhart MK, O’Brien MJ, and Downs SM (1997). Uptake and salvage of hypoxanthine mediates developmental arrest in preimplantation mouse embryos. Biol. Reprod. 56, 1–13. 10.1095/biolreprod56.1.1. 9002627

[51] Gordon RB, Thompson L, Johnson LA, and Emmerson BT (1979). Regulation of purine de novo synthesis in cultured human fibroblasts: the role of P-ribose-PP. Biochim. Biophys. Acta 562, 162–176. 10.1016/0005-2787(79)90135-7. 435498

[52] Kusada H, Hanada S, Kamagata Y, and Kimura N (2014). The effects of N-acylhomoserine lactones, beta-lactam antibiotics and adenosine on biofilm formation in the multi-beta-lactam antibiotic-resistant bacterium Acidovorax sp. strain MR-S7. J. Biosci. Bioeng. 118, 14–19. 10.1016/j.jbiosc.2013.12.012. 24480749

[53] Crane JK, and Shulgina I (2009). Feedback effects of host-derived adenosine on enteropathogenic Escherichia coli. FEMS Immunol. Med. Microbiol. 57, 214–228. 10.1111/j.1574-695X.2009.00598.x. 19751218 PMC2818178

[54] Sheng L, Pu M, Hegde M, Zhang Y, Jayaraman A, and Wood TK (2012). Interkingdom adenosine signal reduces Pseudomonas aeruginosa pathogenicity. Microb. Biotechnol. 5, 560–572. 10.1111/j.1751-7915.2012.00338.x. 22414222 PMC3815332

[55] Fries N (1949). Effects of Different Purine Compounds on the Growth of Guanine-Deficient Ophiostoma. Physiol. Plantarum 2, 78–102. 10.1111/j.1399-3054.1949.tb07651.x.

[56] Brooke MS, and Magasanik B (1954). The metabolism of purines in Aerobacter aerogenes: a study of purineless mutants. J. Bacteriol. 68, 727–733. 10.1128/jb.68.6.727-733.1954. 13221549 PMC386220

[57] Moyed HS (1964). Inhibition of the Biosynthesis of the Pyrimidine Portion of Thiamine by Adenosine. J. Bacteriol. 88, 1024–1029. 10.1128/jb.88.4.1024-1029.1964. 14219014 PMC314849

[58] Traut TW (1994). Physiological concentrations of purines and pyrimidines. Mol. Cell. Biochem. 140, 1–22. 10.1007/BF00928361. 7877593

[59] Sharma K, Zhang G, Hansen J, Bjornstad P, Lee HJ, Menon R, Hejazi L, Liu JJ, Franzone A, Looker HC, (2023). Endogenous adenine mediates kidney injury in diabetic models and predicts diabetic kidney disease in patients. J. Clin. Invest. 133, e170341. 10.1172/JCI170341. 37616058 PMC10575723

[60] Pearson T, Damian K, Lynas RE, and Frenguelli BG (2006). Sustained elevation of extracellular adenosine and activation of A1 receptors underlie the post-ischaemic inhibition of neuronal function in rat hippocampus in vitro. J. Neurochem. 97, 1357–1368. 10.1111/j.1471-4159.2006.03823.x. 16696848

[61] Lan J, Lu H, Samanta D, Salman S, Lu Y, and Semenza GL (2018). Hypoxia-inducible factor 1-dependent expression of adenosine receptor 2B promotes breast cancer stem cell enrichment. Proc. Natl. Acad. Sci. USA 115, E9640–E9648. 10.1073/pnas.1809695115. 30242135 PMC6187157

[62] Chen JF, Eltzschig HK, and Fredholm BB (2013). Adenosine receptors as drug targets–what are the challenges? Nat. Rev. Drug Discov. 12, 265–286. 10.1038/nrd3955. 23535933 PMC3930074

[63] Fleiszig SM, Zaidi TS, Fletcher EL, Preston MJ, and Pier GB (1994). Pseudomonas aeruginosa invades corneal epithelial cells during experimental infection. Infect. Immun. 62, 3485–3493. 10.1128/iai.62.8.3485-3493.1994. 8039920 PMC302982

[64] Kumar NG, Nieto V, Kroken AR, Jedel E, Grosser MR, Hallsten ME, Mettrucio MME, Yahr TL, Evans DJ, and Fleiszig SMJ (2022). Pseudomonas aeruginosa Can Diversify after Host Cell Invasion to Establish Multiple Intracellular Niches. mBio 13, e027422. 10.1128/mbio.02742-22. PMC9765609 36374039

[65] Kimura Y, Turner JR, Braasch DA, and Buddington RK (2005). Lumenal adenosine and AMP rapidly increase glucose transport by intact small intestine. Am. J. Physiol. Gastrointest. Liver Physiol. 289, G1007–G1014. 10.1152/ajpgi.00085.2005. 16020657

[66] Huang Z, Xie N, Illes P, Di Virgilio F, Ulrich H, Semyanov A, Verkhratsky A, Sperlagh B, Yu SG, Huang C, and Tang Y (2021). From purines to purinergic signalling: molecular functions and human diseases. Signal Transduct. Targeted Ther. 6, 162. 10.1038/s41392-021-00553-z. PMC8079716 33907179

[67] Nolan AC, Zeden MS, Kviatkovski I, Campbell C, Urwin L, Corrigan RM, Gründling A, and O’Gara JP (2023). Purine Nucleosides Interfere with c-di-AMP Levels and Act as Adjuvants To Re-Sensitize MRSA To β-Lactam Antibiotics. mBio 14, e02478–e02422. 10.1128/mbio.02478-22. 36507833 PMC9973305

[68] Jacobs MA, Alwood A, Thaipisuttikul I, Spencer D, Haugen E, Ernst S, Will O, Kaul R, Raymond C, Levy R, (2003). Comprehensive transposon mutant library of Pseudomonas aeruginosa. Proc. Natl. Acad. Sci. USA 100, 14339–14344. 10.1073/pnas.2036282100. 14617778 PMC283593

[69] Rietsch A, Vallet-Gely I, Dove SL, and Mekalanos JJ (2005). ExsE, a secreted regulator of type III secretion genes in Pseudomonas aeruginosa. Proc. Natl. Acad. Sci. USA 102, 8006–8011. 10.1073/pnas.0503005102. 15911752 PMC1142391

[70] Simon R, Priefer U, and Pühler A (1983). A Broad Host Range Mobilization System for In Vivo Genetic Engineering: Transposon Mutagenesis in Gram Negative Bacteria. Bio/Technology 1, 784–791. 10.1038/nbt1183-784.

[71] Hoang TT, Kutchma AJ, Becher A, and Schweizer HP (2000). Integration-proficient plasmids for Pseudomonas aeruginosa: site-specific integration and use for engineering of reporter and expression strains. Plasmid 43, 59–72. 10.1006/plas.1999.1441. 10610820

[72] Coppens L, and Lavigne R (2020). SAPPHIRE: a neural network based classifier for sigma70 promoter prediction in Pseudomonas. BMC Bioinf. 21, 415. 10.1186/s12859-020-03730-z. PMC7510298 32962628

[73] Hoang TT, Karkhoff-Schweizer RR, Kutchma AJ, and Schweizer HP (1998). A broad-host-range Flp-FRT recombination system for site-specific excision of chromosomally-located DNA sequences: application for isolation of unmarked Pseudomonas aeruginosa mutants. Gene 212, 77–86. 10.1016/s0378-1119(98)00130-9. 9661666

[74] Ommen P, Zobek N, and Meyer RL (2017). Quantification of biofilm biomass by staining: Non-toxic safranin can replace the popular crystal violet. J. Microbiol. Methods 141, 87–89. 10.1016/j.mimet.2017.08.003. 28802722

[75] Schneider CA, Rasband WS, and Eliceiri KW (2012). NIH Image to ImageJ: 25 years of image analysis. Nat. Methods 9, 671–675. 10.1038/nmeth.2089. 22930834 PMC5554542

