# Supplementary material for: Environmental purines decrease Pseudomonas aeruginosa biofilm formation by disrupting c-di-GMP metabolism
Source: Cell Rep. Author manuscript; Available in PMC 2024 Jun 25. (PMC11197132; doi:10.1016/j.celrep.2024.114154)
Supplement: 1 [file NIHMS2000422-supplement-1.pdf]

Cell Reports, Volume *43*

Supplemental information

Environmental purines decrease *Pseudomonas aeruginosa* biofilm formation by disrupting c-di-GMP metabolism

Corey Kennelly, Peter Tran, and Arthur Prindle

Supplementary Table 1: Oligonucleotides

Primer name	Primer sequence (5' to 3')
PA1543-5	CCTGTCGCTGACCGTGGA
PA1543-3	GGTGAAGTAGCCGCCGTAGAC
PA1543-5-1	GCATAAATGTAAAGCAGGTGATCCTTTCGGCTCGGTC
PA1543-5-2	*AACTCGAGCCGCAAGCATGCTGAA*CGACTTGAGGGTGAACTCGTC
PA1543-3-1	*TTCAGCATGCTTGCGGCTCGAGTT*GCTCTCGACGAGCGTTGATACG
PA1543-3-2	CTAGAGTCGACCTGCAGAGCATCTGGATGGTCAGGTCTTCG
PA4645-5	GCGGATTTCCTTGCTGCCG
PA4645-3	GATCTGGATCAGTTGCTGCTGCTC
PA4645-5-1	GCATAAATGTAAAGCAGCTTCCAGGGTCTCTTCGTTGC
PA4645-5-2	*AACTCGAGCCGCAAGCATGCTGAA*GAGATCGACGGACATGGTGCTTC
PA4645-3-1	*TTCAGCATGCTTGCGGCTCGAGTT*GCTACTGGCGCAACGCCGC
PA4645-3-2	CTAGAGTCGACCTGCAGAGCCAGACTGACGAACAGAAGCAGGGAG
PA5298-5	CTGATCCATCCGCAGCACCTG
PA5298-3	CAACTTCGTCGCCCAGGCTC
PA5298-5-1	GCATAAATGTAAAGCAGGCTGTTGCGTCACATCCAC
PA5298-5-2	*AACTCGAGCCGCAAGCATGCTGAA*GTCTTTGAGAATGTCCACGGGTGG
PA5298-3-1	*TTCAGCATGCTTGCGGCTCGAGTT*CTGCTGGAAAGCGAAGGCTACC
PA5298-3-2	CTAGAGTCGACCTGCAGAACTTGCGTGACATGGTGGTGAC
PA0934-5	GGTGACTGGCAACTGACTCTGG
PA0934-3	CTCGTCACCCTCTGCCATCG
PA0934-5-1	GCATAAATGTAAAGCAGCGGGTCCTCGACCTGTTC
PA0934-5-2	*AACTCGAGCCGCAAGCATGCTGAA*CGCTCTCACCTGTACCATCTTGC
PA0934-3-1	*TTCAGCATGCTTGCGGCTCGAGTT*CTGCCCAACATCATCGAGGCG
PA0943-3-2	CTAGAGTCGACCTGCAGAGACAGGAACGTCATCCAGCAGC
PA5338-5	CTTCATCCTCCCGCCGAGC
PA5338-3	CAGCTTGCCGTCCTTGCTC
PA5338-5-1	GCATAAATGTAAAGCACCGTCAGCGAAATGAGCCACTAC
PA5338-5-2	*AACTCGAGCCGCAAGCATGCTGAA*CAGTCTGTCGGCGAAGGCG
PA5338-3-1	*TTCAGCATGCTTGCGGCTCGAGTT*CTCAAGGGAGTGATCCGAATCACC
PA5338-3-2	CTAGAGTCGACCTGCAGAGGCTGCGGCGAGGAAAATC
PA3770-5	GAGCAGCTCACCGATGTCGTG
PA3770-3	GTGGAATTGCACGCCGTAGTAGG
PA3770-5-1	GCATAAATGTAAAGCAGACGGTAATGTCGTCGTGGCTG
PA3770-5-2	*AACTCGAGCCGCAAGCATGCTGAA*CAGGGCTTCTTGACTGATTCGCAG
PA3770-3-1	*TTCAGCATGCTTGCGGCTCGAGTT*GCCGAGTCCCATGTCCACG
PA3770-3-2	CTAGAGTCGACCTGCAGACTTGTCGCCGTGGCTCATCC
PA1524-5	CGAGCTGATCTGGTGGTACGC
PA1524-3	CGATGACGATGCGGTTGAAGGAAAC
PA1524-5-1	GCATAAATGTAAAGCACCTTCACCCTGCTGGAGATCG
PA1524-5-2	*AACTCGAGCCGCAAGCATGCTGAA*GATCTCCCGGTTGAGCAGGAAC
PA1524-3-1	*TTCAGCATGCTTGCGGCTCGAGTT*CCCGCCGTCGAAACCCG
PA1524-3-2	CTAGAGTCGACCTGCAGACCGAGGAAATCTGCGTCTCCAGGTAG
PA4645-CompF	GGCTGCAGGAATTCGATATCAGGCGACTGTCTCCAACGGG
PA4645-CompR	TCGAGGTCGACGGTATCGATATCAGAGCCCCTTGACCGC
attB-5	GCTGACTTCACGCTGTTCCG
attB-3	GCTACCTGGACTGGGAGTTCG

For PA1543 (apt), PA4645 (hgpt), PA5298 (xpt), PA0934 (relA), PA5338 (spoT), PA3770 (guaB), PA1524 (xdhA), and attB, -5 and -3 indicate primers used to amplify these loci for PCR confirmation and sequencing; -5-1 and -5-2 were used to amplify the region upstream of each gene with appropriate Gibson assembly overhangs while -3-1 and -3-2 were used to amplify the region downstream of each gene with appropriate Gibson assembly overhangs. Within each knockout primer, green font indicates homology with pEXG2; red font with italics indicates arbitrary homology created between -5-2 and -3-1 primers to facilitate Gibson assembly of pEXG2 constructs; black font indicates homology with PAO1 genome; yellow-highlighted font indicates homology with gene of interest. For CompF/R primers, green font indicates homology with pminiCTX; black font indicates homology with PAO1 genome. Grey highlight signifies linker for Gibson assembly.

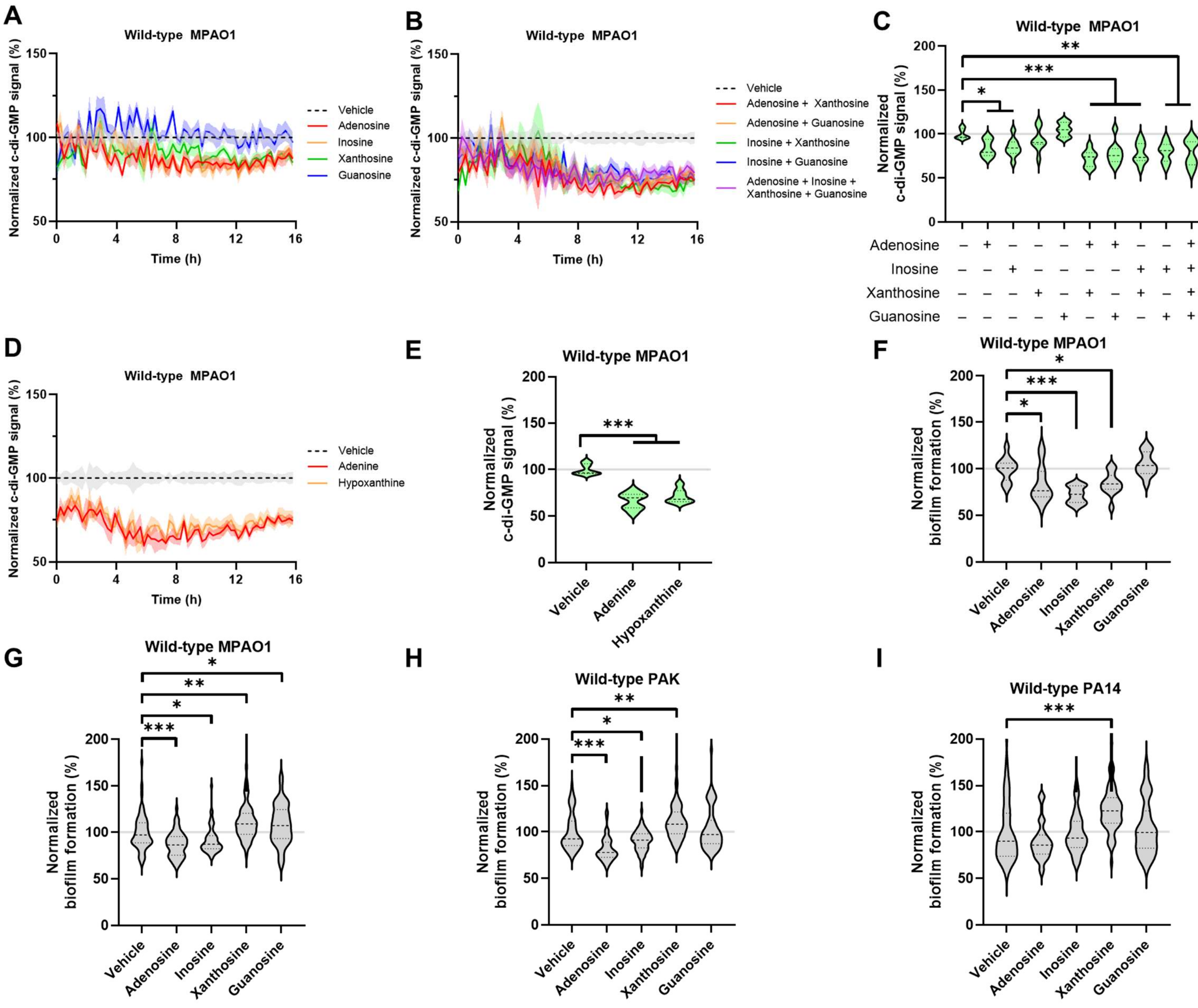

Supplementary Figure 1: Adenosine and inosine decrease c-di-GMP and biofilm formation. (A) c-di-GMP signal time course plots for MPAO1 exposed to vehicle or 100 µM adenosine, inosine, xanthosine, or guanosine. Data are used in Figure 1d, Supplementary Figure 1c, and Supplementary Figure 3a. 3 wells per condition per experiment from 3 independent experiments were included (n=9), barring outliers removed due to aberrant growth: n=8 (Vehicle, Xanthosine). Data represent mean ± SE. **(B)** c-di-GMP signal time course plots for MPAO1 exposed to vehicle or 100 µM combinations of compounds as indicated. Data are used in Figure 1d, Supplementary Figure 1c, and Supplementary Figure 3a. 3 wells per condition per experiment from 3 independent experiments were included (n=9), barring outliers removed due to aberrant growth: n=8 (Vehicle, Adenosine-Inosine-Xanthosine-Guanosine). Data represent mean ± SE. **(C)** Violin plot of c-di-GMP signal for MPAO1 exposed to vehicle or 100 µM indicated compounds after 8 hours of exposure at 37°C, shaking, to quantify changes in Supplementary Figure 1a,b. Data are used in Figure 1d. **(D)** c-di-GMP signal time course plots for MPAO1 exposed to vehicle or 100 µM adenine or hypoxanthine. 3 wells per condition per experiment from 3 independent experiments were included (n=9), barring outliers removed due to aberrant growth: n=8 (Vehicle); n=7 (Hypoxanthine). Data represent mean ± SE. **(E)** Violin plot of c-di-GMP signal for MPAO1 exposed to vehicle or 100 µM adenine or hypoxanthine after 8 hours of exposure at 37°C, shaking, to quantify changes in Supplementary Figure 1d. All c-di-GMP data in Figure 1 and Supplementary Figure 1 were collected simultaneously. **(F)** Violin plots of biofilm formation from safranin-stained biomass at OD_{530} normalized to OD_{600} growth for MPAO1 exposed to vehicle or 100 µM indicated compounds after 8 hours of exposure. In these experiments, biofilms were allowed to grow in 96-well plates at 37°C for 18 hours prior to compound exposure. 3 wells per condition per experiment from 4 independent experiments were included (n=12). **(G)** Violin plots of biofilm formation from safranin-stained biomass at

OD_{530} normalized to OD_{600} growth for *P. aeruginosa* exposed to vehicle or 100 µM indicated compounds after 8 hours of exposure. 9 wells per condition per experiment from 7 independent experiments were included (n=63) unless otherwise noted. Strain MPAO1 was used and n=61 (Vehicle, Adenosine, Inosine, Guanosine) and n=60 (Xanthosine) wells were included after removal of outliers. Biofilm data in Supplementary Figure 1g, 1h, and 1i were collected simultaneously. **(H)** Violin plots of biofilm formation from safranin-stained biomass at OD_{530} normalized to OD_{600} growth for *P. aeruginosa* exposed to vehicle or 100 µM indicated compounds after 8 hours of exposure. 9 wells per condition per experiment from 7 independent experiments were included (n=63) unless otherwise noted. Strain PAK was used and n=62 (Adenosine, Xanthosine, Guanosine) and n=61 (Inosine) wells were included after removal of outliers. Biofilm data in Supplementary Figure 1g, 1h, and 1i were collected simultaneously. **(I)** Violin plots of biofilm formation from safranin-stained biomass at OD_{530} normalized to OD_{600} growth for *P. aeruginosa* exposed to vehicle or 100 µM indicated compounds after 8 hours of exposure. 9 wells per condition per experiment from 7 independent experiments were included (n=63) unless otherwise noted. Strain PA14 was used and n=62 (Xanthosine) wells were included after removal of outliers. Biofilm data in Supplementary Figure 1g, 1h, and 1i were collected simultaneously. Throughout this figure, repeated measures one-way ANOVA with Dunnett's multiple comparison test comparing to vehicle was used to determine statistical significance. *$p \leq 0.05$; **$p \leq 0.01$; ***$p \leq 0.001$.

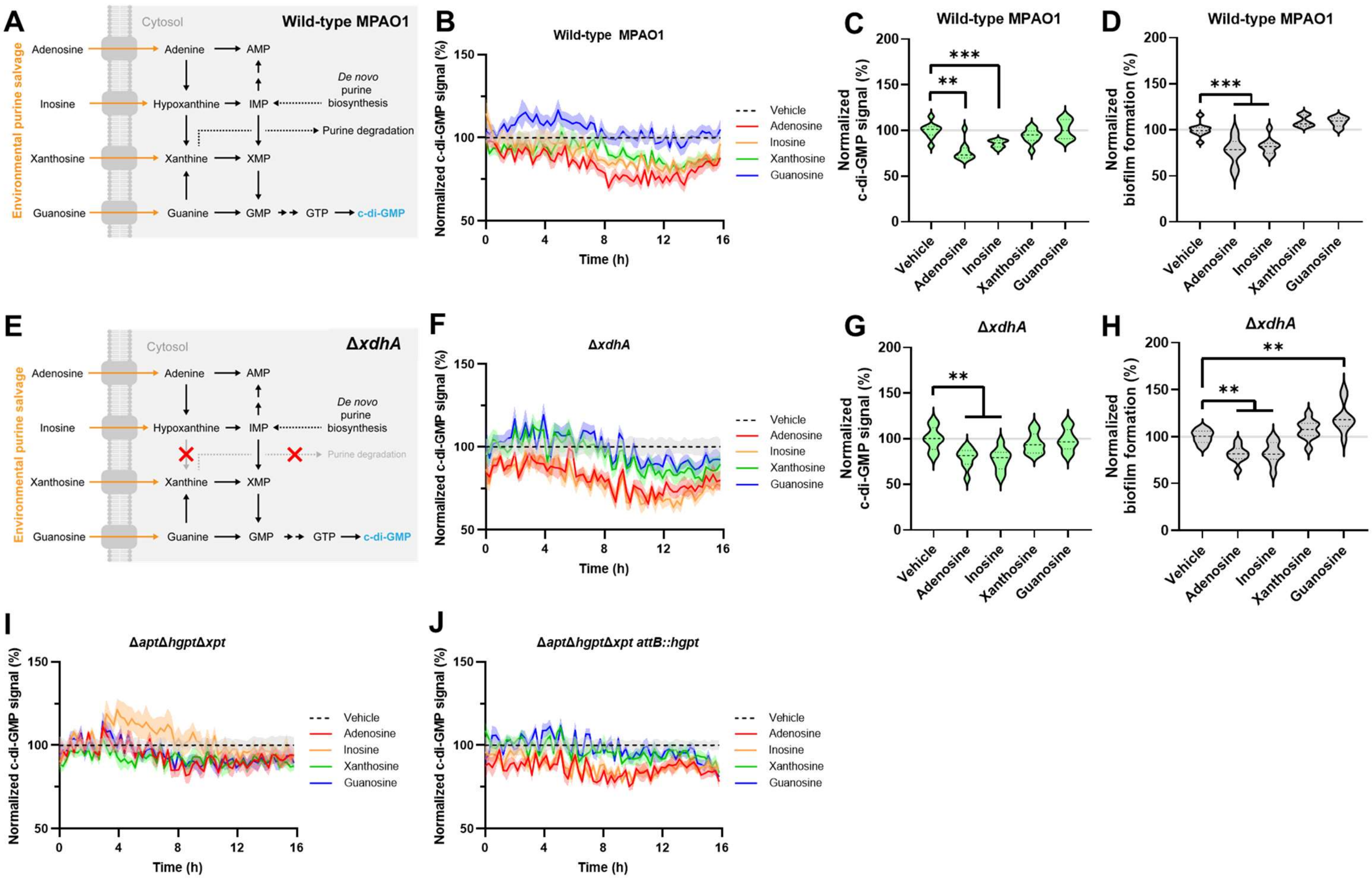

Supplementary Figure 2: Adenosine/inosine-dependent effects require purine salvage. (A) MPAO1 can import environmental purines and use them in both salvage and degradation pathways. **(B)** c-di-GMP signal time course plots for MPAO1 exposed to vehicle or 100 µM adenosine, inosine, xanthosine, or guanosine. 3 wells per condition per experiment from 3 independent experiments were included (n=9), barring an outlier removed due to aberrant growth: n=8 (Inosine). Data represent mean ± SE. **(C)** Violin plot of c-di-GMP signal for MPAO1 exposed to vehicle or 100 µM adenosine, inosine, xanthosine, guanosine after 8 hours of exposure at 37°C, shaking, to quantify changes in Supplementary Figure 2b. **(D)** Violin plot of biofilm formation from safranin-stained biomass for MPAO1 exposed to vehicle or 100 µM adenosine, inosine, xanthosine, or guanosine after 8 hours of exposure. 3 wells per condition per experiment from 3 independent experiments were included (n=9). **(E)** Δ*xdhA* can import environmental purines and use them in the salvage pathway but not the degradation pathway. **(F)** c-di-GMP signal time course plots for Δ*xdhA* exposed to vehicle or 100 µM adenosine, inosine, xanthosine, or guanosine. 3 wells per condition per experiment from 3 independent experiments were included (n=9), barring outliers removed due to aberrant growth: n=8 (Vehicle, Inosine). Data represent mean ± SE. **(G)** Violin plot of c-di-GMP signal for Δ*xdhA* exposed to vehicle or 100 µM adenosine, inosine, xanthosine, guanosine after 8 hours of exposure at 37°C, shaking, to quantify changes in Supplementary Figure 2f. **(H)** Violin plot of biofilm formation from safranin-stained biomass for Δ*xdhA* exposed to vehicle or 100 µM adenosine, inosine, xanthosine, or guanosine after 8 hours of exposure. 3 wells per condition per experiment from 3 independent experiments were included (n=9). **(I)** c-di-GMP signal time course plots for Δ*apt*Δ*hgpt*Δ*xpt* exposed to vehicle or 100 µM adenosine, inosine, xanthosine, or guanosine. 3 wells per condition per experiment from 3 independent experiments were included (n=9), barring outliers removed due to aberrant growth: n=8 (Adenosine, Inosine, Guanosine); n=7 (Xanthosine). Data are used in Figure 2b. Data represent mean ± SE. **(J)** c-di-GMP signal time course plots for Δ*apt*Δ*hgpt*Δ*xpt attB::hgpt* exposed to vehicle or 100 µM adenosine, inosine, xanthosine, or guanosine. 3 wells per condition per experiment from 3 independent experiments were included (n=9), barring outliers removed due to aberrant growth: n=7 (Adenosine). Data are used in Figure 2e. Data represent mean ± SE. All c-di-GMP data in Figure 2 and Supplementary Figure 2 were collected simultaneously. All biofilm data in Figure 2 and Supplementary Figure 2 were collected simultaneously. Throughout this figure, repeated measures one-way ANOVA with Dunnett's multiple comparison test comparing to vehicle was used to determine statistical significance. *p≤0.05; **p≤0.01; ***p≤0.001.

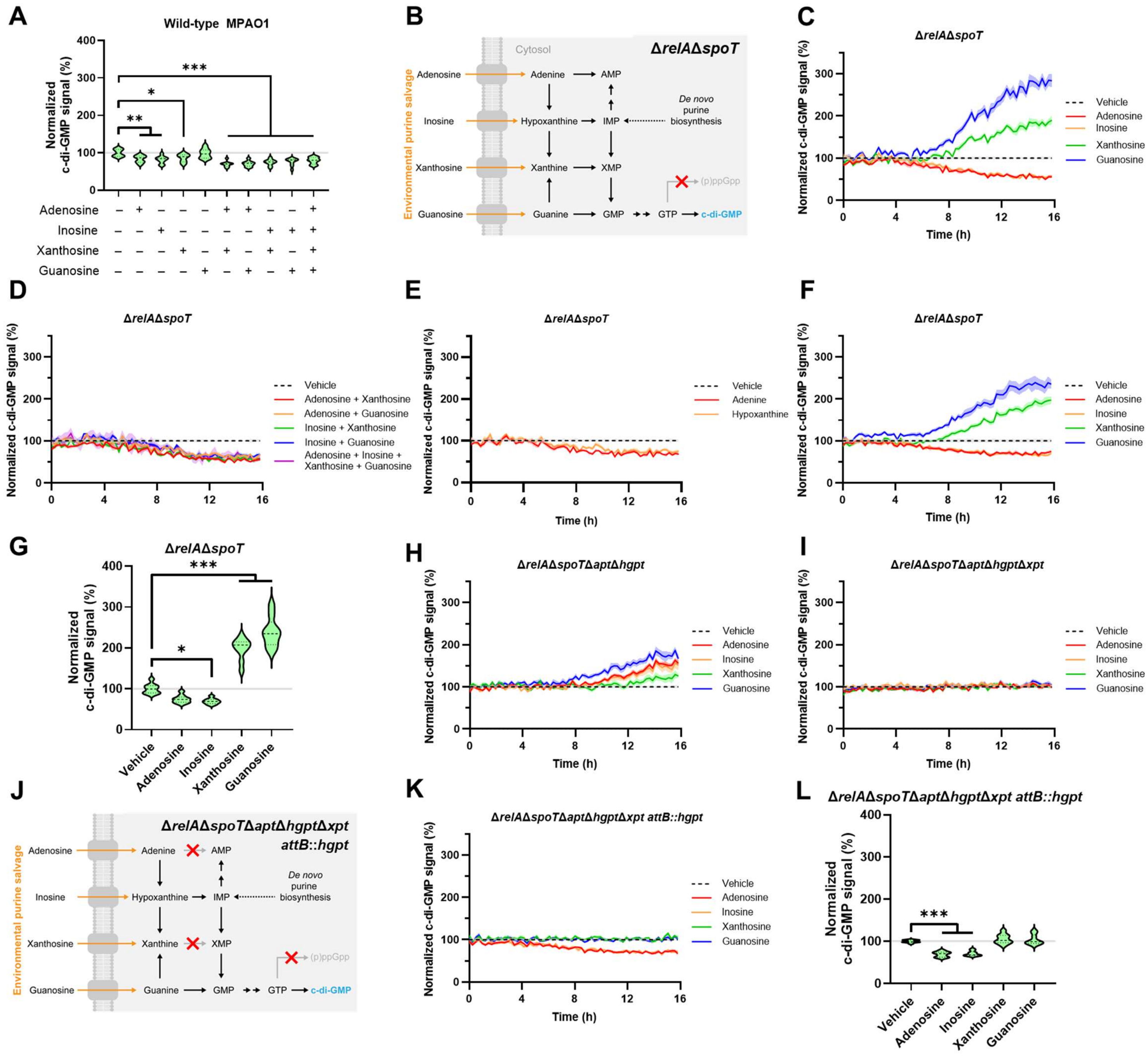

Supplementary Figure 3: (p)ppGpp prevents xanthosine/guanosine-dependent effects and their salvage is also blocked by adenosine and inosine. (A) Violin plot of c-di-GMP signal for MPAO1 exposed to vehicle or 100 µM indicated compounds after 12 hours of exposure at 37°C, shaking. 3 wells per condition per experiment from 3 independent experiments were included (n=9), barring outliers removed due to aberrant growth: n=8 (Vehicle, Xanthosine). Data are from Supplementary Figure 1a,b. **(B)** Δ*relA*Δ*spoT* is capable of salvaging all purines; however, extracellular purines may affect this strain differently compared to MPAO1 due to the absence of (p)ppGpp, a negative regulator of GTP homeostasis in some bacteria. **(C)** c-di-GMP signal time course plots for Δ*relA*Δ*spoT* exposed to vehicle or 100 µM adenosine, inosine, xanthosine, or guanosine. 3 wells per condition per experiment from 3 independent experiments were included (n=9), barring outliers removed due to aberrant growth: n=8 (Vehicle). Data was collected simultaneously with Supplementary Figure 1a,b,d. Data are used in Figure 3b. Data represent mean ± SE. **(D)** c-di-GMP signal time course plots for Δ*relA*Δ*spoT* exposed to vehicle or 100 µM combinations of compounds as indicated. 3 wells per condition per experiment from 3 independent experiments were included (n=9), barring outliers removed due to aberrant growth: n=8 (Vehicle, Xanthosine+Adenosine, Xanthosine+Inosine); n=7 (Guanosine+Adenosine); n=5 (Adenosine+Inosine+Xanthosine+Guanosine). Data was collected simultaneously with Supplementary Figure 1a,b,d. Data are used in Figure 3b. Data represent mean ± SE. **(E)** c-di-GMP signal time course plots for Δ*relA*Δ*spoT* exposed to

vehicle or 100 µM adenine or hypoxanthine. 3 wells per condition per experiment from 3 independent experiments were included (n=9), barring outliers removed due to aberrant growth: n=8 (Vehicle, Hypoxanthine). Data was collected simultaneously with Supplementary Figure 1a,b,d. Data represent mean ± SE. **(F)** c-di-GMP signal time course plots for Δ*relA*Δ*spoT* exposed to vehicle or 100 µM adenosine, inosine, xanthosine, or guanosine. 3 wells per condition per experiment from 3 independent experiments were included (n=9). Data was collected simultaneously with Supplementary Figure 3h,i,k. Data represent mean ± SE. **(G)** Violin plot of c-di-GMP signal for Δ*relA*Δ*spoT* exposed to vehicle or 100 µM adenosine, inosine, xanthosine, guanosine after 16 hours of exposure at 37°C, shaking, to quantify changes in Supplementary Figure 3f. **(H)** c-di-GMP signal time course plots for Δ*relA*Δ*spoT*Δ*apt*Δ*hgpt* exposed to vehicle or 100 µM adenosine, inosine, xanthosine, or guanosine. 3 wells per condition per experiment from 3 independent experiments were included (n=9), barring outliers removed due to aberrant growth: n=7 (Xanthosine). Data was collected simultaneously with Supplementary Figure 3f, i, k. Data are used in Figure 3d. Data represent mean ± SE. **(I)** c-di-GMP signal time course plots for Δ*relA*Δ*spoT*Δ*apt*Δ*hgpt*Δ*xpt* exposed to vehicle or 100 µM adenosine, inosine, xanthosine, or guanosine. 3 wells per condition per experiment from 3 independent experiments were included (n=9). Data was collected simultaneously with Supplementary Figure 3f, h, k. Data are used in Figure 3f. Data represent mean ± SE. **(J)** (p)ppGpp-mediated GTP homeostasis remains absent but intracellular nucleotide pools are no longer insulated from environmental purines in Δ*relA*Δ*spoT*Δ*apt*Δ*hgpt*Δ*xpt attB::hgpt* background due to expression of *hgpt* from native promoter at the *attB* neutral site. **(K)** c-di-GMP signal time course plots for Δ*relA*Δ*spoT*Δ*apt*Δ*hgpt*Δ*xpt attB::hgpt* exposed to vehicle or 100 µM adenosine, inosine, xanthosine, or guanosine. 3 wells per condition per experiment from 3 independent experiments were included (n=9). Data was collected simultaneously with Supplementary Figure 3f, h, i. Data represent mean ± SE. **(L)** Violin plot of c-di-GMP signal for Δ*relA*Δ*spoT*Δ*apt*Δ*hgpt*Δ*xpt attB::hgpt* exposed to vehicle or 100 µM adenosine, inosine, xanthosine, guanosine after 16 hours of exposure at 37°C, shaking, to quantify changes in Supplementary Figure 3k. Throughout this figure, repeated measures one-way ANOVA with Dunnett's multiple comparison test comparing to vehicle was used to determine statistical significance. *p≤0.05; **p≤0.01; ***p≤0.001.

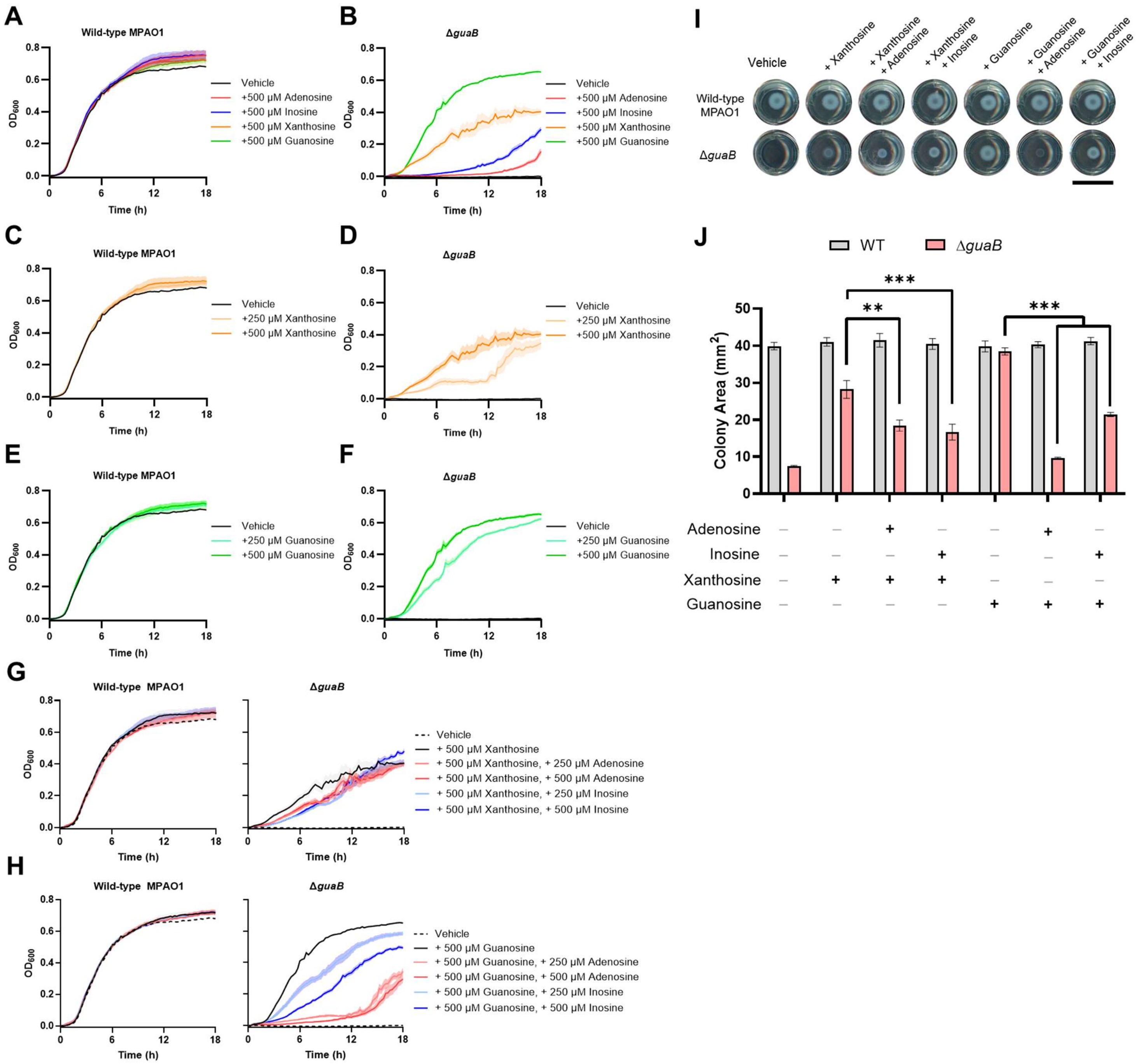

Supplementary Figure 4: Guanine auxotroph reveals adenosine and inosine block guanosine-dependent effects even at expense of growth. (A) Growth of MPAO1 in M9 supplemented with vehicle or 500 µM of adenosine, inosine, xanthosine, or guanosine, respectively, at 37°C, shaking. 3 wells per condition per experiment from 3 independent experiments were included (n=9). **(B)** Growth of Δ*guaB* in M9 supplemented with vehicle or 500 µM of adenosine, inosine, xanthosine, or guanosine, respectively, at 37°C, shaking. 3 wells per condition per experiment from 3 independent experiments were included (n=9). **(C)** Growth of MPAO1 in M9 supplemented with vehicle, 250 µM, or 500 µM xanthosine, respectively, at 37°C, shaking. 3 wells per condition per experiment from 3 independent experiments were included (n=9). **(D)** Growth of Δ*guaB* in M9 supplemented with vehicle, 250 µM, or 500 µM xanthosine, respectively, at 37°C, shaking. 3 wells per condition per experiment from 3 independent experiments were included (n=9), barring an outlier removed due to aberrant growth: n=8 (Δ*guaB* 500 µM Guanosine, 500 µM Inosine). **(E)** Growth of MPAO1 in M9 supplemented with vehicle, 250 µM, or 500 µM guanosine, respectively, at 37°C, shaking. 3 wells per condition per experiment from 3 independent experiments were included (n=9). **(F)** Growth of Δ*guaB* in M9 supplemented with vehicle, 250 µM, or 500 µM guanosine, respectively, at 37°C, shaking. 3 wells per condition per experiment from 3 independent experiments were included (n=9). **(G)** Growth of MPAO1 and Δ*guaB* in M9 supplemented with vehicle or indicated mixtures of compounds

including xanthosine at 37°C, shaking. 3 wells per condition per experiment from 3 independent experiments were included (n=9). **(H)** Growth of MPAO1 and Δ*guaB* in M9 supplemented with vehicle or indicated mixtures of compounds including guanosine at 37°C, shaking. 3 wells per condition per experiment from 3 independent experiments were included (n=9), barring an outlier removed due to aberrant growth: n=8 (Δ*guaB* 500 μM guanosine, 500 μM inosine). **(I)** Image of MPAO1 and Δ*guaB* growth on M9 agar pads supplemented with vehicle or 300 μM of indicated compounds at 37°C after 24 hours. 2 wells per condition per experiment from 4 independent experiments were included for vehicle, guanosine, adenosine-guanosine, inosine-guanosine, and xanthosine conditions (n=8). 1 well per condition per experiment from 4 independent experiments were included for adenosine-xanthosine and inosine-xanthosine experiments (n=4). Scale indicates 15 mm. A subset of this data is displayed in Figure 4c. **(J)** Bar plot of MPAO1 and Δ*guaB* colony size from Supplementary Figure 4i quantified using ImageJ. All data represent mean ± SE. Repeated measures one-way ANOVA with Dunnett's multiple comparison test comparing to supplemented xanthosine or supplemented guanosine condition was used to determine statistical significance. **p≤0.01; ***p≤0.001.
